# Modulating immune cell fate and inflammation through CRISPR-mediated DNA methylation editing

**DOI:** 10.1126/sciadv.adt1644

**Published:** 2025-07-16

**Authors:** Gemma Valcárcel, Aleksey Lazarenkov, Anna V. López-Rubio, Clara Berenguer, Josep Calafell-Segura, Javier Rodríguez-Ubreva, Esteban Ballestar, José Luis Sardina

**Affiliations:** ^1^ Epigenetic Control of Haematopoiesis Group, Josep Carreras Leukaemia Research Institute (IJC), Campus Can Ruti, 08916 Badalona, Spain.; ^2^ Doctoral Program in Biomedicine, Universitat de Barcelona (UB), 08036 Barcelona, Spain.; ^3^ Epigenetics and Immune Disease Group, Josep Carreras Leukaemia Research Institute (IJC), Campus Can Ruti, 08916 Badalona, Spain.; ^4^ Epigenetics in Inflammatory and Metabolic Diseases Laboratory, Health Science Center (HSC), East China Normal University (ECNU), Shanghai 200241, China.

## Abstract

Immune cell differentiation and activation are associated with widespread DNA methylation changes; however, the causal relationship between these changes and their impact in shaping cell fate decisions still needs to be fully elucidated. Here, we conducted a genome-wide analysis to investigate the relationship between DNA methylation and gene expression at gene regulatory regions in human immune cells. By using CRISPR-dCas9-TET1 and -DNMT3A epigenome editing tools, we successfully established a cause-and-effect relationship between the DNA methylation levels of the promoter of the interleukin-1 receptor antagonist (*IL1RN*) gene and its expression. We observed that modifying the DNA methylation status of the *IL1RN* promoter is sufficient to alter human myeloid cell fate and change the cellular response to inflammatory and pathogenic stimuli. Collectively, our findings demonstrate the potential of targeting specific DNA methylation events to directly modulate immune and inflammatory responses, providing a proof of principle for intervening in a broad range of inflammation-related diseases.

## Teaser

*IL1RN* DNA methylation editing alters myeloid cell fate and immune responses, revealing epigenetic drivers of immune diseases.

## INTRODUCTION

DNA methylation (DNAm) at CpG dinucleotides is the main epigenetic modification of mammalian DNA and is commonly associated with transcriptional repression (*1*). This link arises from extensive research in the fields of differentiation and development, investigating the effects on transcription of disrupting the function of key DNAm enzymes, such as DNA methyltransferases (DNMTs) and Ten-Eleven Translocation methylcytosine dioxygenases (TETs) (*2*–*4*). In cancer research, drugs such as azacitidine and decitabine, DNA hypomethylating agents, have been widely used to investigate the connection between DNAm and transcription (*5*, *6*). Both approaches offer valuable insights, but they have the limitation of altering the overall methylome of the cells, posing a challenge in distinguishing between direct and indirect effects on gene expression. In addition to their catalytic activity, DNMTs and TETs can interact with transcription- and chromatin-related factors, thus affecting transcription through mechanisms independent of DNAm (*7*–*12*). Furthermore, DNMTs and TETs, which have antagonistic roles, have been described as requiring each other to develop their genome-wide catalytic activity properly (*13*, *14*), adding another layer of complexity to the interpretation of the loss-of-function experiments. Last, small molecules with hypomethylating effects have diverse biological functions unrelated to DNAm (*15*). Therefore, the extent to which DNAm directly instructs gene expression and cell fate remains unclear.

A better understanding of the instructive role of DNAm in transcriptional outcomes has been recently achieved by using tools that allow precise addition or removal of DNAm marks (*6*, *16*, *17*), especially when coupled with single-cell and quantitative readouts (*18*). Such studies have revealed context-specific transcriptional responses associated with DNAm gain at promoter regions, including increased gene expression (*16*). These unexpected responses may result from the varying sensitivity of different transcription factors (TFs) to the presence of DNAm at their specific recognition sites in the genome (*19*) or the DNAm’s ability to counteract the polycomb-mediated deposition of H3K27me3 (*20*).

The interplay between TFs and epigenetic regulators is considered essential during hematopoiesis, substantially shaping immune cell identity and function (*21*). However, the causal connections between DNAm and immune cell fates have not been thoroughly explored. Pioneer editing studies demonstrated that targeted DNAm editing could stably silence gene expression in immune cells (*22*). More recently, CRISPR-based DNAm editing in human hematopoietic stem cells led to altered differentiation outcomes (*23*). Despite these important advances, a deeper understanding of the molecular mechanisms that drive changes in immune identity following DNAm editing is urgently needed, given its critical implications for blood cell differentiation and cancer. In addition, comprehensive studies addressing the causal relationship between DNAm and relevant immune responses, such as inflammation, are still lacking in relevant models of immune cell fate identity and function.

In our study, we investigated how changes in DNAm at gene regulatory regions affect gene expression during the reprogramming of human leukemic B cells into non-tumorigenic macrophages (*24*). These DNAm changes resembled those observed when comparing human primary B cells with primary macrophages, including changes at the *IL1RN* promoter. Therefore, we used CRISPR-dCas9-TET1 and -DNMT3A editing tools in the cellular model to precisely alter the DNAm status of the *IL1RN* promoter and evaluate its impact on relevant immune cellular phenotypes. Our findings revealed that IL1RN contributes functionally to the acquisition of myeloid cell identity in humans. In addition, we demonstrated that the methylation-edited cells exhibited altered responses to both inflammatory and pathogenic stimuli, including enhanced secretion of proinflammatory cytokines and an altered ability to support cancer cell proliferation upon interleukin-1β (IL-1β) treatment. These results highlight the potential of DNAm editing to directly modulate immune and inflammatory responses, providing a direct mechanistic link between epigenetic remodeling and immune-related disease phenotypes.

## RESULTS

### WGBS uncovers demethylation of myeloid-related GREs during human B-to-macrophage reprogramming

To gain insight into the dynamics of DNAm during myeloid cell fate acquisition, we chose to use the highly efficient and homogenous CCAAT/enhancer binding protein α (C/EBPα)-driven conversion of human B leukemic cells, containing a β-estradiol inducible form of C/EBPα (BlaER cells), into non-tumorigenic induced macrophages (iMacs) (*24*). The iMacs generated using this protocol closely resemble their natural counterparts as they are fully phagocytic and have inflammasome competency (*24*–*26*).

**Fig. 1.**
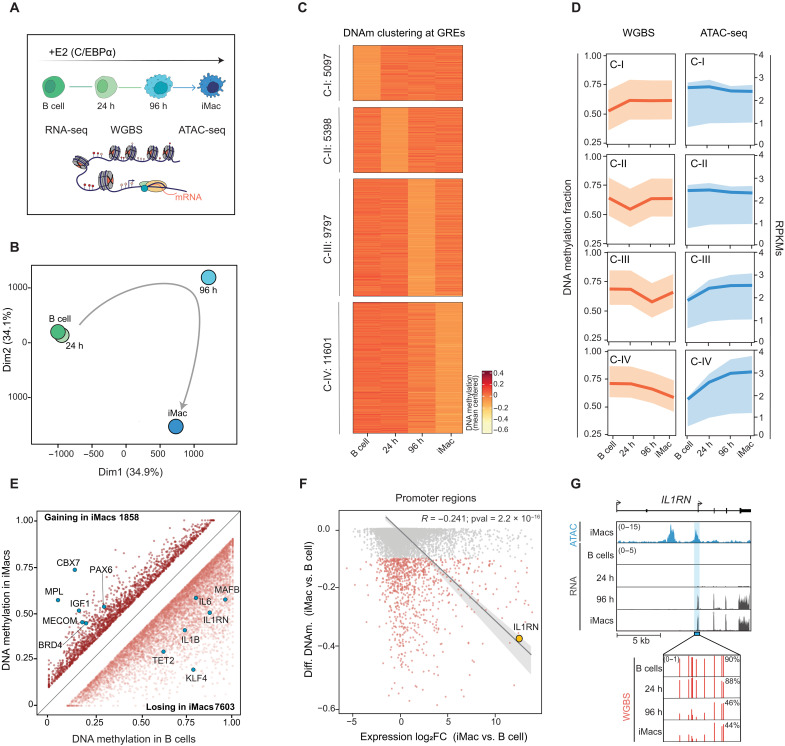
Integrative epigenome analyses uncover *IL1RN* promoter as a top DNAm-expression correlated event during myeloid cell commitment. (**A**) Schematic overview of samples and methodology used in the epigenome profiling. iMac, induced macrophages. (**B**) Multidimensional Scaling analysis showing DNAm dynamics at 1-kb bins genome-wide during transdifferentiation. A gray arrow indicates the hypothetical trajectory. (**C**) Clustering of genome-wide DNAm dynamics (by WGBS) at 1-kb bins at GREs. Only 1-kb bins showing at least 10% DNAm changes are depicted. (**D**) Quantification of DNAm levels (by WGBS) and chromatin accessibility (by ATAC-seq) at the clusters in (C). Plots represent the mean (line) and interquartile range (shaded region). (**E**) Scatter plot comparing DNAm levels at iMac’s chromatin accessible regions between iMacs and B cells (only ΔDNAm>10% are shown). Blue dots highlight ATAC^+^ regions of interest. (**F**) Plot showing the correlation between the step changes (iMac versus B cells) in DNAm and gene expression at iMac’s chromatin accessible regions (ATAC^+^ peaks). Light red dots, promoter regions losing at least 10% of DNAm in iMacs. The large yellow dot highlights the correlation between DNAm and expression at the *IL1RN* promoter. (**G**) Genome browser snapshot showing signal for chromatin accessibility (by ATAC-seq) in iMacs, and gene expression (by RNA-seq) during transdifferentiation at the *IL1RN* locus. The blue-shaded region represents the DNAm dynamic promoter of the *IL1RN* short isoform (ENST00000409930.4). h, hours.

Cultured B cells were treated with β-estradiol (E2) and collected at different time points of the reprogramming process (0 hours, B cells; 24, 96, and 168 hours, iMacs) (Fig. 1A). We then generated genome-wide, nucleotide-resolution maps for DNAm using whole-genome bisulfite sequencing (WGBS). DNAm levels were determined by computing the values of approximately 24 million CpG residues covered ≥5× in all the samples. After induction of C/EBPα, a genome-wide reshaping of DNAm was observed from 96 hours onward (Fig. 1B and fig. S1A). Approximately 14% of the DNAm signal, which accounts for 384,27 1-kb bins, was redistributed during this process (fig. S1B). The redistribution of the DNAm signal did not occur randomly in the genome; instead, it occurred preferentially on specific chromosomes (fig. S1B).

To further understand the genomic context where the redistribution of DNAm occurs, we intersected the DNAm dynamic bins (fig. S1B) with Assay for Transposase-Accessible Chromatin (ATAC) using sequencing (ATAC-seq) and H3K4me1 chromatin immunoprecipitation sequencing (ChIP-seq) data collected during the transdifferentiation process (*26*, *27*). Then, we selected bins that contained at least one peak of ATAC associated with a transcription start site (TSS) (promoters) or with an H3K4me1 peak (enhancers). As a result, 31,893 DNAm dynamic bins containing gene regulatory elements (GREs) were identified, accounting for approximately only 8% of all dynamic bins observed. Since GREs are important for cell fate decisions (*28*), we focused all our further DNAm analyses exclusively on them. To achieve this, the approximately 32,000 dynamic DNAm bins containing GREs were grouped into four major clusters (C-I to C-IV) based on the timing of their DNAm changes. The clusters exhibited predominantly sequential and mostly transient changes in DNAm (C-I to C-III), except for cluster IV (Fig. 1C). Cluster IV, containing approximately one-third of the total number of regions, showed continuous loss of DNAm from 96 hours onward (Fig. 1C). Of note, only regions included in clusters showing demethylation from 96 hours onward (C-III and C-IV), regardless of transient or continuous behavior, experienced an increase in chromatin accessibility (by ATAC-seq) during the reprogramming process (Fig. 1D). Conversely, no chromatin closure was observed in association with the gain of DNAm (C-I regions) (Fig. 1D). Thus, suggesting DNAm gain might not regulate chromatin accessibility in this cellular model of myeloid cell fate acquisition. To investigate this hypothesis further, we specifically analyze changes in DNAm at chromatin-accessible regions (ATAC-positive) between B cells and iMacs. In iMacs, we observed 7603 ATAC-positive regions that lost DNAm and 1858 regions that gained it, which were preferentially associated with C-III and C-IV, and C-I and C-II, respectively, as expected (Fig. 1E and fig. S1C). The regions of DNAm loss and gain exhibited different genomic distributions (fig. S1D). The gain regions mainly overlapped with introns and promoters at similar percentages (35.9 and 29.1%, respectively). Conversely, in the loss regions, there was a preferential enrichment in intronic regions, possibly intragenic enhancers, at the expense of promoters (41.8 and 21%, respectively) (fig. S1D). To uncover biological processes potentially regulated by the DNAm changes described, we performed Gene Ontology (GO) analysis of the genes located at the ATAC-positive DNAm loss and gain regions (fig. S1E). This showed enrichment of terms related to myeloid cells for genes associated with loss regions, including “myeloid leukocyte migration,” “phagocytosis,” and “positive regulation of cytokine production,” among others. On the other hand, the gain regions were nonspecifically associated with terms related to organ development, such as “axon guidance,” “kidney development,” and “heart morphogenesis,” among others (fig. S1E). In line with these findings, TF motif analysis showed significant enrichment of TFs relevant for myeloid cell biology, such as AP1 complex subunits (including Fos, JunB, BATF, and Fra1-2, among others) and CEBP factors, in the ATAC-positive DNAm loss regions (fig. S1F). In contrast, the DNAm gain regions displayed a modest enrichment in the motifs of genome architecture TFs, including CTCF and its relative BORIS (also known as CTCFL), along with development-related TFs such as Hox, Slug, or Snail, among others (fig. S1F). Of note, the predicted specific occupancy of C/EBP factors in the regions losing DNAm was validated using C/EBPα ChIP-seq data in B cells and iMacs (fig. S1G).

Together, this suggests that specific GREs related to myeloid cells are undergoing DNA demethylation and potential chromatin activation during the B-to-macrophage reprogramming. At the same time, GREs unrelated to the cell fate conversion process are gaining DNAm without apparent changes in chromatin activity.

### Integrative epigenomic profiling reveals the *IL1RN* promoter as a top event showing a DNAm-expression correlation during transdifferentiation

To gain mechanistic insight into the transcriptional consequences of the DNAm changes observed at GREs, we investigated the interplay between DNAm and gene expression using RNA sequencing (RNA-seq) data (*26*, *27*). We observed no correlation between the gain of DNAm at ATAC-positive regions and changes in gene expression of their associated genes (*R* = −0.018; *P* val. = 0.64) (fig. S1H). In contrast, a significant negative correlation was detected between DNAm loss and gene expression at ATAC-positive regions, both at non-promoter (*R* = −0.132; *P* = 7.2 × 10^−10^) and promoter-overlapping sites, where the association was even stronger (*R* = −0.241; *P* = 2.2 × 10^−16^) (Fig. 1F). Among the top correlated events was the promoter of the IL-1 receptor antagonist (*IL1RN*) gene, which showed more than 40% DNAm loss and more than a 1000-fold increase in expression between iMacs and B cells (Fig. 1F). The *IL1RN* gene, which plays an essential role in inflammatory responses in mature myeloid cells (*29*), is not expressed, and its promoter is fully methylated at the B cell stage (Fig. 1G). However, the methylation of the *IL1RN* promoter decreases between 24 and 96 hours, which coincides with the reactivation of the gene (Fig. 1G). These changes occur specifically at the promoter of the *IL1RN* short isoform, *IL1RN-205* (ENST00000409930.4), corresponding to the secretory protein variant preferentially expressed in myeloid cells (*29*). These analyses suggest that only gene regulatory regions losing DNAm, such as the promoter of the *IL1RN* gene, but not regions gaining it, correlate with gene expression changes during human B-to-macrophage transdifferentiation.

### *IL1RN* promoter emerges as a top DNAm-expression correlated event during myeloid cell fate acquisition in primary human cells

**Fig. 2.**
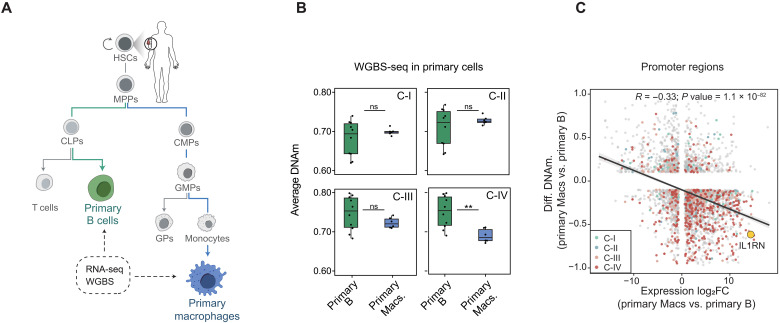
*IL1RN* promoter shows strong methylation–expression correlation during myeloid cell commitment in human primary cells. (**A**) Schematic overview of samples and methodology used in the epigenome profiling of human primary blood cells collected from the Blueprint Consortium. (**B**) DNAm levels (by WGBS) in primary human B cells and primary human macrophages (Macs) at the clusters in (Fig. 1C). Each dot represents an individual sample. Unpaired two-tailed Student’s *t* test (**P < 0.01 *n* = 10 versus *n* = 6). (**C**) Plot showing the correlation between the step changes in DNAm at promoter ATAC^+^ regions and gene expression (primary Macs versus B cells). The colored dots are labeled according to their belonging to clusters in (Fig. 1C). The large yellow dot highlights the *IL1RN* promoter region in primary blood cells. ns, not significant. d, days.

To place our transdifferentiation-based observations into a physiological context, we analyzed WGBS and RNA-seq datasets from the Blueprint Consortium, focusing on primary human B cells and macrophages (Fig. 2A). Notably, among all regions showing DNAm changes during transdifferentiation (Fig. 1, C and D), only those that lost methylation from 96 hours onward, classified as C-IV, exhibited significant methylation differences between primary B cells and macrophages (Fig. 2B). This suggests that these regions may harbor GREs relevant to myeloid fate decisions. Correlation analyses further revealed that C-IV regions were enriched among loci showing both DNAm loss and increased gene expression, accounting for more than 50% of such events (Fig. 2C).

The *IL1RN* promoter ranked among the top regions displaying strong inverse correlation between DNAm and gene expression, supporting its potential physiological relevance in human myeloid differentiation (Fig. 2C). Consistent with this, a pan-hematopoietic analysis revealed that *IL1RN* promoter demethylation and gene activation occur selectively in myeloid cells, further reinforcing its role as a myeloid-specific gene within the hematopoietic system (fig. S2, A to D).

### dCas9-TET1–mediated demethylation of the *IL1RN* promoter is sufficient to lead to its gene reactivation

While the phenotypes associated with the loss of function of major DNAm regulators have been extensively studied (*2*–*4*), the impact of individual methylation events on immune phenotypes remains largely unexplored, mainly due to technical limitations (*5*, *6*). This has been recently challenged with the advent of DNAm editing tools, especially those associated with CRISPR technology (*30*).

**Fig. 3.**
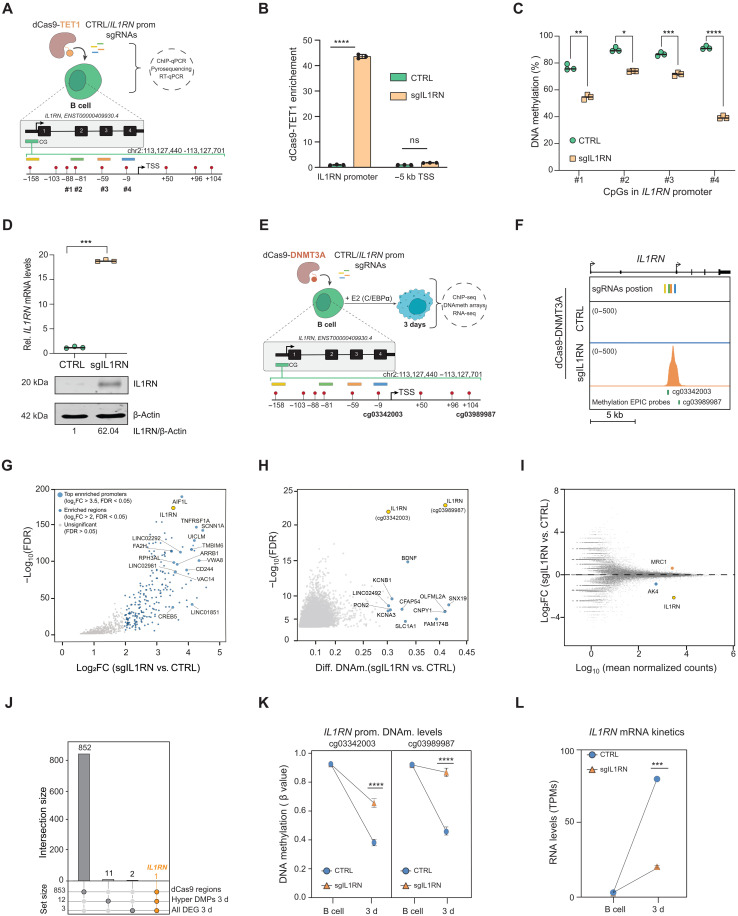
CRISPR-mediated epigenome editing at the *IL1RN* promoter efficiently modulates DNAm levels and gene expression. (**A**) Schematic of the dCas9-TET1 *IL1RN* promoter epigenome editing. The four sgRNAs targeting the *IL1RN* TSS are shown. #1 to #4 indicate the CpGs analyzed in (C). (**B**) ChIP-qPCR showing dCas9-TET1 enrichment at the *IL1RN* promoter. A 5-kb downstream region from the TSS is shown as a negative control region. Unpaired two-tailed Student’s *t* test, *n* = 3, mean SEM, (****P < 0.0001). (**C**) DNAm levels (pyrosequencing) at four CpGs upstream of *IL1RN* TSS [as in (A)] in CTRL and edited B cells. One-way analysis of variance (ANOVA) with Dunnett’s post hoc correction, *n* = 3, means ± SEM, (*P < 0.05; **P < 0.01, ***P<<0.001,****P < 0.0001). (**D**) Top, *IL1RN* expression (RT-qPCR) in CTRL and edited B cells. Unpaired two-tailed Student’s *t* test, *n* = 3, means ± SEM, (***P < 0.001). Bottom, IL1RN protein levels in CTRL and edited B cells. Fold change normalized to β-actin (CTRL versus sgIL1RN). (**E**) Schematic of the dCas9-DNMT3A *IL1RN* promoter epigenome editing experiment as in (A), showing two Infinium MethylationEPIC v2.0 probes. (**F**) Genome browser snapshot showing dCas9-DNMT3A binding at the *IL1RN* promoter. The location of the four sgRNAs targeting the *IL1RN* promoter and the two array probes is depicted. (**G**) Scatter plot showing differential dCas9-DNMT3A enrichment in sgIL1RN B cells. Large dots indicate top-bound promoters. (**H**) Scatter plot showing differentially hypermethylated CpGs in sgIL1RN day-3 cells. Blue dots indicate significantly hypermethylated CpGs (Δβ ≥ 0.3, FDR < 0.05, *n* = 13). Yellow dots highlight *IL1RN* probes shown in (E). (**I**) MA plot showing DEGs in sgIL1RN day-3 cells. (**J**) Upset plot depicting overlap of the dCas9-DNMT3A–binding sites, hypermethylated CpGs, and DEGs. (**K**) DNAm dynamics (array data) of two significantly hypermethylated *IL1RN* promoter CpGs. Unpaired two-tailed Student’s *t* test, *n* = 4, means ± SEM, (****P < 0.0001). (**L**) *IL1RN* dynamics (RNA-seq) in CTRL and edited cells. Unpaired two-tailed Student’s *t* test, *n* = 2, means ± SEM, (***P < 0.001).

In our study, we focused on the *IL1RN* promoter, which ranked as the top region showing a strong DNAm-expression correlation when comparing the cellular model with primary cells (Fig. 1, F and G, and Fig. 2, A to C). In addition, given its myeloid-specific epigenetic regulation (fig. S2, A to D), we selected this region as a target for DNAm editing to explore its potential role in modulating myeloid lineage specification and function. We first hypothesized that inducing DNA demethylation at this region could drive *IL1RN* expression at the B cell stage. To address this challenge, we stably integrated a previously developed DNA demethylation editing tool (*31*, *32*), into our human B leukemic cells (Fig. 3A). The editing tool is based on a catalytically dead Cas9 protein tethered with the catalytic domain of the DNA methylcytosine dioxygenase TET1 (dCas9-TET1). B cell clones expressing high levels of the dCas9-TET1 transgene were selected and expanded. Subsequently, we transduced dCas9-TET1 clones with lentiviruses simultaneously encoding four different single guide RNAs (sgRNAs) that targeted either no sequence within the genome (CTRL cells) or the *IL1RN* promoter (sgIL1RN cells) (Fig. 3A). We confirmed that the dCas9-TET1 protein was specifically bound to the *IL1RN* promoter in sgIL1RN B cells (Fig. 3B). The dCas9-TET1 recruitment led to the demethylation of CpG residues located upstream of the gene’s TSS (Fig. 3C). Among them, the largest demethylation (approximately 50% decrease) was observed at the CpG residue located immediately upstream of the TSS [−9 base pair (bp)] (Fig. 3C). Last, because of the DNA demethylation mediated by dCas9-TET1, we detected *IL1RN* gene activation (>15-fold increase in mRNA levels) and protein accumulation (>60-fold increase) in sgIL1RN B cells (Fig. 3D). dCas9-TET1 editing successfully activated the expression of a myeloid-specific gene (fig. S2, A to D) in an inappropriate immune cell type (B cells), thereby unveiling a causal relationship between the DNAm status of the *IL1RN* promoter and the expression of its gene.

### dCas9-DNMT3A efficiently and specifically hypermethylates the *IL1RN* promoter, leading to its down-regulation

As previously shown, the *IL1RN* promoter is demethylated between 24 and 96 hours of the cell fate conversion process, coinciding with the reactivation of its gene (Fig. 1G). Therefore, on the basis of the observed causality between DNAm and *IL1RN* expression (Fig. 3, C and D), we hypothesized that blocking the DNAm loss associated with the transdifferentiation process would alter *IL1RN* gene expression patterns. To tackle this task, we used a previously developed DNAm editing tool (*31*, *32*) based on a catalytically dead Cas9 protein tethered with the catalytic domain of the DNA de novo methyltransferase DNMT3A (dCas9-DNMT3A). Both the DNAm editing tool and the sgRNAs were integrated into our human B leukemic cells as previously outlined for the dCas9-TET1 experiment (Fig. 3E). We confirmed that the dCas9-DNMT3A protein was strongly bound to the *IL1RN* promoter in sgIL1RN B cells by ChIP-seq (Fig. 3F and fig. S3A). However, as expected from previous research using these DNAm editing tools (*32*–*34*), we also observed the off-target binding of the dCas9-DNMT3A protein to 852 genomic regions (Fig. 3G). Of note, some of the strongest off-target sites (log_2_FC > 3.5) were identified as overlapping promoters. Therefore, the recruitment of the dCas9-DNMT3A protein may result in the hypermethylation of these promoters and the deregulation of their genes. However, in B cells, none of the off-target regions or the *IL1RN* promoter itself (already hypermethylated) showed differential methylation and their associated genes were not differentially expressed (fig. S3, B to F). We then examined the potential methylation and expression impacts of dCas9-DNMT3A off-target binding 3 days after induction, when the *IL1RN* promoter would have started losing methylation. This allowed us to identify differences in the DNAm levels between the control and sgIL1RN cells at this specific region (Fig. 3H). Our findings showed that only 13 CpG residues were hypermethylated, with two of them exhibiting the most significant changes in the *IL1RN* promoter itself (Fig. 3H). In addition, we observed only three differentially expressed genes (DEGs), with *IL1RN* showing more than a fourfold down-regulation in sgIL1RN 3-day cells (Fig. 3I).

Overall, the only region differentially occupied by dCas9-DNMT3A, differentially methylated and differentially expressed when comparing sgIL1RN cells and CTRL cells is the *IL1RN* gene (Fig. 3, J to L, and fig. S3, F and G). Thus, the specificity and accuracy of the dCas9-DNMT3A–mediated DNAm editing were validated, and the previously described causality between methylation and expression at this gene was reinforced.

### dCas9-DNMT3A–mediated hypermethylation of the *IL1RN* promoter leads to impaired human myeloid cell fate acquisition and enhanced phagocytic capacity

The IL-1 pathway has been described as playing a role in maintaining balanced hematopoiesis between the lymphoid and myeloid lineages in mice. Both chronic exposure to IL-1β (*35*) and genetic deficiency of *Il1rn* (*36*) lead to hyperactivation of the IL-1 pathway, which causes a bias toward the myeloid lineage. However, the exact role of IL1RN and the IL-1 pathway in the acquisition of human myeloid cell fate and the molecular mechanisms underlying the myeloid bias following hyperactivation of the IL-1 pathway are not yet fully understood.

**Fig. 4.**
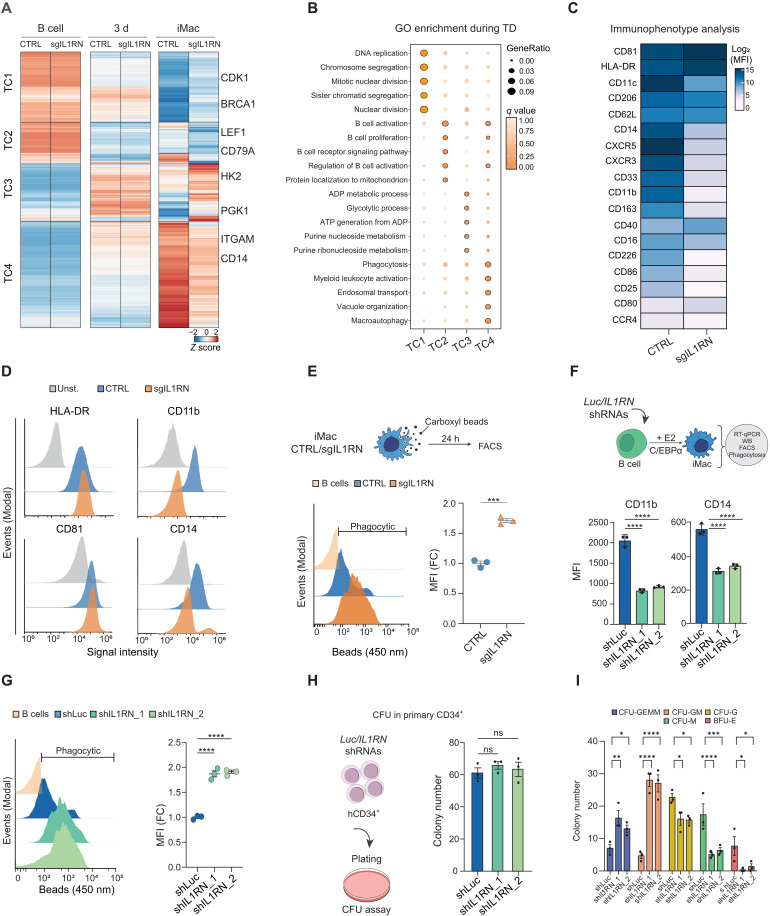
*IL1RN* depletion alters myeloid cell fate and phagocytic function. (**A**) Clustering of the DEGs during transdifferentiation between dCas9-DNMT3A CTRL and *IL1RN* edited cells. (*n* = 2 biologically independent replicates, FDR < 0.05). TC1 to TC4, transdifferentiation clusters. Representative genes for each cluster are annotated. (**B**) Gene Ontology Biological Processes (GO:BP) enrichment analysis for the genes associated with clusters in (A). The top five most significant terms for each cluster are plotted. Significant terms (*q* value < 0.05) are highlighted with a black stroke. (**C**) Mean fluorescence intensity (MFI) of macrophage-lineage surface markers in CTRL and edited iMacs. (**D**) Representative histograms showing signal intensity for selected cell surface markers in CTRL and edited iMacs. (**E**) Phagocytic capacity evaluation [by fluorescence-activated cell sorting (FACS)] for CTRL and edited iMacs. Left, histogram showing the uptake of blue fluorescent beads. Right, blue beads MFI quantification. Unpaired two-tailed Student’s *t* test, *n* = 3, means ± SEM, (***P < 0.001). (**F**) Up, schematic of the *IL1RN* shRNAs experiments during transdifferentiation. Bottom, MFI quantification of myeloid markers in shLuc and shIL1RN iMacs. Two-way ANOVA with Dunnett’s post hoc test, *n* = 3, means ± SEM, (****P < 0.0001). (**G**) Phagocytic capacity evaluation (by FACS) for shLuc and shIL1RN iMacs. Left, histogram showing the uptake of blue fluorescent beads. Right, blue beads MFI quantification. Two-way ANOVA with Dunnett’s post hoc test, *n* = 3, means ± SEM, (****P < 0.0001). (**H**) Left, schematic of the colony-forming assay performed in shLuc and shIL1RN bone marrow–derived human CD34^+^ cells; CFU, colony forming unit. Right, total number of CFUs obtained from shLuc and shIL1RN_1/_2 CD34^+^ cells. *n* = 3 biologically independent samples per group, means ± SEM. (**I**) Comparison of different types of CFUs obtained from shLuc and shIL1RN_1/_2 CD34^+^ cells. Two-way ANOVA with Dunnett’s post hoc test, *n* = 3, means ± SEM,(*P < 0.05; **P < 0.01; ****P < 0.0001).

The kinetics of *IL1RN* expression during our human B-to-macrophage reprogramming suggest a potential involvement of IL1RN and the IL-1 pathway in the physiological acquisition of the human myeloid cell fate. To explore this possibility, we analyzed the transcriptomic profiles of CTRL and sgILRN cells throughout the transdifferentiation process. As previously observed, minor changes in gene expression were detected at short time points (fig. S4A). This is expected since *IL1RN* expression is detected from day 3 onward (Fig. 3, I and L). At the iMac stage, a distinct transcriptional profile emerged between the CTRL and sgILRN cells, with 3795 DEGs [false discovery rate (FDR) < 0.05] (fig. S4, A and B). Among these, 1752 genes were up-regulated, and 2043 were down-regulated. One of the down-regulated genes was *IL1RN* itself, which showed a strong reduction of about 16-fold in its mRNA levels (fig. S4B) and was largely decreased at the protein level in dCas9-DNMT3A sgIL1RN iMacs (fig. S4C). To investigate further the impact of *IL1RN* DNAm editing on the transcriptomic kinetics, all the genes differentially expressed in dCas9-DNMT3A CTRL and sgILRN cells throughout the transdifferentiation process were selected and clusterized into four major groups [transdifferentiation clusters (TC) 1 to 4] (Fig. 4A). As expected, differences in gene expression within the clusters between CTRL and sgIL1RN cells were only detectable at the iMac stage (Fig. 4A and fig. S4D). In TC1, the *IL1RN* DNAm editing led to an incomplete silencing of genes related to chromosome segregation, such as the well-known *BRCA1* or *CDK1* genes (Fig. 4B and fig. S4D). In TC2, *IL1RN* DNAm editing further enhanced the down-regulation of genes involved in the B cell transcriptional program, including the genes coding for the B cell TF *LEF1* and the marker *CD79A* (Fig. 4B and fig. S4D). In TC3, the DNAm editing prevented the decrease in gene expression for a group of transiently up-regulated genes related to metabolic processes, including hexokinase 2 (*HK2*) and phosphoglycerate kinase 1 (*PGK1*) (Fig. 4B and fig. S4D). Last, in TC4, the DNAm editing hindered the up-regulation of genes related to myeloid cell fate and phagocytosis processes, including *ITGAM* and *CD14* (Fig. 4B and fig. S4D). Together, these transcriptional analyses suggest that modifying the methylation status of the *IL1RN* promoter could influence the cell fate acquired during the transdifferentiation process, leading to cells with an altered cellular division, metabolism, and myeloid differentiation status.

To support the notion that IL1RN depletion leads to an altered myeloid cell fate, we performed extensive immunoprofiling of 18 monocyte/macrophage lineage surface markers using spectral flow cytometry. We compared the alterations in CTRL- and sgIL1RN-iMacs, along with human monocyte-derived macrophages differentiated in vitro in the presence of either granulocyte-macrophage colony-stimulating factor (GM-CSF) or M-CSF (fig. S4E). We observed marker-specific similarities between GM-CSF– or M-CSF–differentiated macrophages and CTRL-iMacs, for instance, with GM-CSF for human leukocyte antigen (HLA)–DR and M-CSF for CD11b (fig. S4, F and G). Notably, *IL1RN* editing broadly reduced myeloid marker levels, with 14 of 18 markers exhibiting lower protein abundance (Fig. 4C and fig. S4G), including general myeloid markers CD11b (encoded by *ITGAM*) and CD14 (Fig. 4D). This suggested an overall disruption in myeloid cell fate acquisition following *IL1RN* methylation editing, which was further supported by the observation that edited cells had an enhanced phagocytic capacity (Fig. 4E).

Together, we have shown the involvement of IL1RN in the establishment of the C/EBPα-driven myeloid cell fate and acquisition of enhanced phagocytic capacity. Thus, this suggests that the IL-1 pathway may be necessary for the proper acquisition of myeloid cell fate during human hematopoiesis.

### shRNA-mediated *IL1RN* depletion impairs primary myeloid cell fate commitment and alters the transdifferentiation phenotypes

To validate the results obtained with the dCas9-DNMT3A tool, we used an orthogonal method. We infected our human B leukemic cells with lentiviruses harboring short hairpin RNAs (shRNAs) that target the *IL1RN* mRNA. We used shRNAs against the firefly luciferase (Luc) as a control (Fig. 4F). Both shRNAs efficiently depleted IL1RN at the mRNA and protein levels in iMacs (fig. S4, H and I). The *IL1RN*-depleted (shIL1RN) iMacs displayed similar changes in gene expression as observed in *IL1RN* DNAm–edited iMacs, including increased levels of genes related to cell division (*BRCA1* and *CDK1*) and metabolism (*HK2* and *PGK1*), as well as reduced levels of B- (*LEF1* and *CD79A*) and myeloid- (*ITGAM* and *CD14*) related genes (fig. S4J). Furthermore, the shIL1RN iMacs also exhibited reduced levels of the myeloid surface markers CD11b and CD14 and enhanced phagocytic capacity compared with control iMacs (Fig. 4, F and G).

To gain physiological insights into IL1RN’s role in myeloid cell commitment and differentiation, we performed colony-forming assays using human bone marrow–derived CD34^+^ cells transduced with control or *IL1RN*-targeting shRNAs (Fig. 4H and fig. S4K). While a nonsignificant trend toward increased total colony numbers was observed in sh*IL1RN* conditions, IL1RN-depleted CD34^+^ cells showed a marked reduction in the formation of mature myeloid colonies (Fig. 4, H and I). Instead, there was a significant shift toward less differentiated colony types, such as granulocyte-erythroid-macrophage-megakaryocyte (GEMM) and granulocyte-macrophage progenitors (GMPs) (Fig. 4, H and I). These results, obtained using an orthogonal approach, not only reinforce our epigenome editing findings but also extend them by providing evidence that IL1RN, and likely the IL-1 pathway, regulates human myeloid cell fate in both the transdifferentiation model and primary human myeloid differentiation assays.

### C/EBPα binding to key IL-1 pathway–related GREs coincides with their activation during the acquisition of the myeloid cell fate

On the basis of our observation of IL1RN’s dependency on the correct acquisition of the myeloid cell fate in our cellular model (Fig. 4, A to G, and fig. S4, A to J) and in the differentiation of primary human CD34^+^ cells (Fig. 4, H and I), we analyzed its epigenomic regulation to gain insight into its transcriptional control. We observed that C/EBPα, which drives the transdifferentiation process and is essential for myeloid commitment in mice (*37*), binds to the *IL1RN* promoter as early as 24 hours after induction (fig. S5A). This binding occurs before the DNA demethylation of the region and the transcriptional activation (Figs. 1G and 3K), but it temporally coincides with a substantial epigenome rewiring, as indicated by increased chromatin accessibility and activation (detected by ATAC-seq and H3K27ac decoration, respectively) at the C/EBPα-bound region (fig. S5A).

The *IL1B* locus was also found to undergo demethylation during the transdifferentiation process (Fig. 1E). This GRE is a proximal enhancer (PE) of the gene (PE2 at −5.5 kb from the TSS) that is bound by C/EBPα along with its promoter and yet another PE (PE1 at −3 kb from the TSS) as early as 24 hours of transdifferentiation (fig. S5B). In B cells, both the promoter and the PE1 of the *IL1B* gene are already accessible and devoid of DNAm (fig. S5B). However, the binding of C/EBPα to the PE2 occurs in a closed chromatin region that only becomes accessible after its binding, coinciding with the loss of DNAm in the region (fig. S5B). The C/EBPα binding to the *IL1B* promoter, PE1 and PE2 regions might finally favor the establishment of a temporary super-enhancer, which is observed to cover the PEs and the entire gene body (approximately 10 kb). This is indicated by the coverage of the whole region with H3K4me1 and H3K27ac, ultimately leading to increased transcriptional output, as shown by the enhanced activation of the promoter (by H3K4me3 deposition) (fig. S5B). Notably, these epigenetic changes at the *IL1B* locus are accompanied by the full processing and release of IL-1β into the supernatant, indicating that an active inflammasome complex is operating in these cells during the transdifferentiation process (fig. S5, C and D).

The observed DNA demethylation and chromatin activation events at the *IL1RN* and *IL1B* genes upon C/EBPα binding to their GREs strongly suggest that this TF plays an important role in controlling the IL-1 pathway activity during the establishment of the myeloid cell fate. To gain insight into the physiological process, we studied the transition from mouse hematopoietic stem progenitor cells (HSPCs) to GMPs, a process driven by C/EBPα (*37*). We only observed chromatin activation at the *Il1rn* and *Il1b* loci at the GMP stage, specifically at the C/EBPα-bound regions, coinciding with the GREs described in the human cellular cell fate model (fig. S5, E and F). Together, these analyses reveal a previously unidentified mechanistic role for C/EBPα as a potentially crucial transcriptional regulator of the IL-1 pathway during both human and murine myeloid cell fate determination.

### *IL1RN* DNAm editing modifies the early transcriptional response to IL-1β, leading to enhanced p65/NF-κB and diminished IFN pathway activation

**Fig. 5.**
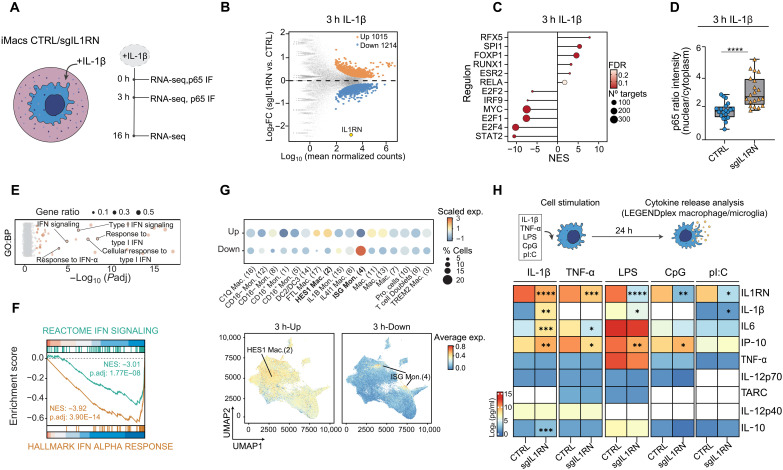
*IL1RN* methylation editing disrupts NF-κB/IFN activation by IL-1β and dampens macrophage responses to immune challenges. (**A**) Schematic of the IL-1β treatment experiment in CTRL and sgIL1RN edited iMacs. (**B**) MA plot showing DEGs in CTRL and sgIL1RN iMacs treated with IL-1β for 3 hours. (**C**) Lollipop plot depicting the DoRothEA TF activity predicted in CTRL and edited iMacs treated with IL-1β for 3 hours. (**D**) Quantification of p65 nuclear versus cytoplasmic localization signal in CTRL and edited iMacs treated with IL-1β for 3 hours. Unpaired two-tailed Student’s *t* test, *n* = 20 cells per group, means ± SEM, (****P < 0.0001). (**E**) GO:BP enrichment analysis of the most significantly down-regulated genes (log_2_FC > 0.5, FDR < 0.05) at 3 hours of IL-1β treatment. Significant terms are shown in orange. (**F**) GSEA of IFN signature in edited iMacs treated with IL-1β for 3 hours. NES, normalized enrichment score. (**G**) Expression profiling of the 3-hour Up and 3-hour Down gene signatures in (B) across the MoMac-Verse scRNA-seq dataset (*40*). Top, average gene signatures scaled expression across the identities. Bottom, Uniform Manifold Approximation and Projection (UMAP) plots showing the average expression of each gene signatures. Cell identities with the highest signature expression are bolded and shown in the UMAPs. (**H**) Top, schematic representation of the cytokine release assay in CTRL and sgIL1RN iMacs, in response to various inflammatory and pathogenic stimuli. Bottom, heatmap of the cytokines concentrations in response to different stimuli in the iMacs. White rectangles represent cytokine concentrations below the detection threshold of the assay. Unpaired two-tailed Student’s *t* test with Benjamini-Hochberg, *n* = 6 biologically independent samples per group, means ± SEM, (*P < 0.05; **P < 0.01; ***P < 0.001; ****P < 0.0001).

Since IL1RN acts as a negative regulator of the IL-1 signaling pathway (*38*), the *IL1RN* DNAm–edited (sgIL1RN) iMacs should exhibit an enhanced response to an inflammatory stimulus mediated by IL-1β, the pathway agonist. To characterize this, we assessed the transcriptomic profiles of sgIL1RN and CTRL iMacs either untreated (0 hours) or treated with IL-1β for 3 or 16 hours (Fig. 5A and fig. S6A). We observed large transcriptomic changes at 0 hours (2212 DEGs, FDR < 0.05) and 3 hours (2229 DEGs, FDR < 0.05) of IL-1β treatment, which diminished following prolonged exposure (166 16-hour DEGs, FDR < 0.05) (Fig. 5B and fig. S6, B to D). To further characterize the potential impact of the transcriptional differences observed at 0 and 3 hours between sgIL1RN and CTRL iMacs, we used DoRothEA (Discriminant Regulon Expression Analysis). DoRothEA is a manually curated human regulon database used to estimate the activities of TFs in individual samples based on the expression of their target genes (*39*). The analysis showed higher activity in regulons related to important myeloid TFs such as SPI1 (PU.1) or RUNX1, and the inflammation-related TF RELA/(p65), as well as lower activity in regulons associated with the interferon (IFN) pathway [signal transducers and activators of transcription 2 (STAT2) or IFN regulatory factor 9 (IRF9)] in sgIL1RN compared to CTRL iMacs, both at 0 hours and mostly at 3 hours after IL-1β treatment (Fig. 5C and fig. S6E). In line with the predicted RELA/(p65) increased transcriptional activity, we observed enhanced p65 nuclear localization in sgIL1RN iMacs at 0 and 3 hours of the IL-1β treatment (Fig. 5D and fig. S6, F and G). This was supported by the up-regulation, at 3 hours, of approximately 50 bona fide p65 target genes, including *NFKBIA*, *NFKB2*, *JUN*, or *CD74* (fig. S6H). In addition, GO and Gene Set Enrichment Analyses (GSEA) showed clear enrichments for biological processes and signaling pathways related to the IFN response in the down-regulated genes in *IL1RN*-edited macrophages (Fig. 5, E and F). This was further supported by the marked down-regulation, at 3 hours, of more than 50 IFN-associated genes, including *ISG15*, *USP18*, *IFIT3,* and members of the *OAS* gene family (fig. S6I).

Therefore, these analyses suggest that methylation editing at the *IL1RN* promoter alters the activation balance between two key inflammatory pathways: the p65/nuclear factor κB (NF-κB) pathway and the IFN pathway. Notably, the potential cross-talk between IL1RN and the NF-κB pathway has been previously described in the context of acute myeloid leukemia (AML) cells (*36*). However, our findings extend this understanding by suggesting that IL1RN may also modulate IFN signaling, highlighting its broader role in orchestrating interactions between inflammatory pathways in myeloid cells.

### *IL1RN* DNAm editing alters the myeloid transcriptional activation fate in response to IL-1β

To understand the physiological relevance of the observed differential transcriptional response to the IL-1β treatment between sgIL1RN and CTRL iMacs, we examined a publicly available dataset that displays the transcriptional profiles of 17 different monocyte and macrophage identities at single-cell resolution (MoMac-VERSE) (fig. S6J) (*40*). We first analyzed the levels of *IL1RN* to determine which cellular identities might depend on its function. Our analysis revealed that *IL1RN* is preferentially expressed in the CD16-negative monocyte (CD16^−^Mon., 12), the IL1B monocyte (IL1B Mon., 15), and the IFN Stimulating Gene monocyte (ISG Mon., 4) identities (fig. S6K). Then, we assessed the expression of the 0- and 3-hour DEGs detected between sgIL1RN and CTRL iMacs (Fig. 5B and fig. S6C) across the 17 identities contained in the MoMac-VERSE single-cell RNA sequencing (scRNA-seq) dataset. Notably, the up-regulated genes, which are more expressed in the sgIL1RN macrophages, are mainly expressed in CD16-negative monocytes (CD16- Mon., 8) and HEIS1 macrophages (HEIS1 Mac., 2). Conversely, the down-regulated genes, which are more expressed in CTRL macrophages, are preferentially expressed in ISG-Mon (4) identity (Fig. 5G and fig. S6L). Notably, this aligns with the observed enrichment of the IFN signature and associated genes in the 3-hour down-regulated subset (Fig. 5, E and F, and fig. S6I). Our analysis suggests that the differential transcriptional response to IL-1β in *IL1RN*-depleted cells may have physiological relevance and provides evidence supporting the idea that *IL1RN* methylation editing alters myeloid cell fate activation.

### *IL1RN* DNAm editing alters the late response to IL-1β, enhancing the IFN pathway over p65/NF-κB activation

As anticipated on the basis of the transcriptional kinetics observed in response to IL-1β, the differences between sgIL1RN and CTRL iMacs were no longer apparent after 16 hours of IL-1β treatment (fig. S6, B and D). At this point, the transcriptional activity we detected with DoRothEA was the mirror image of the patterns observed at 0 and 3 hours. Specifically, we noticed higher activity in regulons associated with the IFN pathway, such as STAT2 or IRF9, and lower activity in regulons related to the NF-κB transcriptional complex, such as REL, RELA, or NFKB1 (fig. S6M). This may be due to the prolonged stimulation of the IL-1 pathway resulted in the activation of feedback inhibitory loops previously described to operate in this pathway for resolving inflammation (*41*). To further characterize this observation, we investigated the impact of *IL1RN* DNAm editing on the IL-1β–induced transcriptomic kinetics. We identified genes differentially expressed between sgILRN and CTRL iMacs during IL-1β treatment and categorized them into four major clusters (IL-1β clusters, IC- 1 to IC-4). Regardless of the initial transcriptional status or the early response to IL-1β treatment, genes in all clusters exhibited no differences after 16 hours of IL-1β treatment (fig. S7, A and B). In IC-1, *IL1RN* DNAm editing led to impaired down-regulation of genes related to lipopolysaccharide (LPS) response or phagocytosis, as exemplified by *CD14* (fig. S7, B and C). In IC-2, *IL1RN* DNAm editing induced a strong and transient gene up-regulation at 3 hours of IL-1β treatment of genes related to metabolic biological processes, such as the mitochondrially encoded ATP synthase 6, *MT-ATP6* (fig. S7, B and C). IC-3 contains genes that exhibit reduced expression at 0 hours and impaired up-regulation at 3 hours. These genes are enriched in viral response biological processes, exemplified by the type I IFN–induced gene 2′-5′-oligoadenylate synthetase 3 (*OAS3*) (fig. S7, B and C). Last, IC-4 contains genes involved in DNA recombination, such as *IL7R*, which show reduced expression in *IL1RN* DNAm–edited cells at 0 hours and reach CTRL levels at 3 hours of IL-1β treatment (fig. S7, B and C). The enrichment of IC-3 genes in viral response GO terms coincides with the observation that down-regulated genes are enriched in IFN-related biological processes and pathways and preferentially expressed by ISG monocytes (4) (Fig. 5, E to G, and fig. S6I). Therefore, we investigated the transcriptional signatures of the dynamic clusters across the MoMac-VERSE scRNA-seq dataset. As expected, we found a preferential expression of the IC-3 signature in ISG monocytes (5), as demonstrated by the exclusive expression pattern of *OAS3* (fig. S7, D and E).

We have shown that altering the methylation status of the *IL1RN* promoter influences how cells respond to the inflammatory stimulus induced by IL-1β. This alteration disrupts the balance between the activation of the NF-κB and IFN signaling pathways over time following exposure to IL-1β, potentially resulting in modified cellular responses. In addition, we have identified ISG monocytes as the primary monocyte-macrophage population that may be substantially affected by the disruption of *IL1RN* levels.

### *IL1RN* promoter methylation rewires macrophage cytokine responses to inflammatory and pathogenic stimuli

The altered transcriptional response observed in sgIL1RN macrophages following IL-1β stimulation at both early and late phases (Fig. 5 and figs. S6 and S7) suggested that *IL1RN* editing might broadly affect the capacity of the iMacs to respond to external inflammatory and pathogenic stimuli. To test this, we challenged CTRL and sgIL1RN macrophages with IL-1β, tumor necrosis factor–α (TNF-α), LPS, CpG, and pI:C, and assessed their response by profiling the secretion of 10 cytokines (Fig. 5H and fig. S8A). As anticipated, IL-1β stimulation elicited the most pronounced changes, significantly altering the release of 5 of the 10 cytokines tested, including well-known proinflammatory cytokines, such as IL-1β, IL-6, and the IFN-γ–induced protein 10 (IP-10). However, significant alterations in cytokine secretion were observed across all conditions, with at least two cytokines affected per stimulus. Notably, IL-1RN secretion was consistently dysregulated across all five tested conditions, indicating a pervasive effect of *IL1RN* editing regardless of the nature of the stimulus (Fig. 5H and fig. S8A). In addition, IP-10 secretion was significantly altered in response to all treatments except pI:C, while IL-1β release was affected following stimulation with IL-1β, LPS, and pI:C (Fig. 5H and fig. S8A). The most consistently altered cytokines across different stimuli—IL-1RN, IL-1β, and IP-10—are encoded by genes that are highly expressed in the ISG-Mon (4) and IL1B-Mon (15) populations, as identified in the scRNA-seq MoMac-Verse dataset (fig. S8B). This observation links the altered cytokine response to a transcriptional signature characteristic of ISG and IL1B monocytes, suggesting that *IL1RN* editing may shift macrophage identity toward these inflammatory subtypes. We observed that the expression levels of these cytokine coding genes were significantly up-regulated in lung and colon cancer ISG and IL1B monocytes compared to their healthy counterparts (fig. S8C). This suggests that *IL1RN* editing may drive macrophages toward an inflammatory transcriptional program associated with ISG and IL1B monocyte identities, which is further amplified in the tumor context. To assess whether *IL1RN* editing alters the tumor-modulatory potential of the macrophage secretome, we collected supernatants from CTRL and sgIL1RN iMacs after 24 hours of IL-1β stimulation, which were differentially enriched in IL-1RN, IL-1β, and IP-10 (fig. S8D). Colon (HCT116) and lung (Cal-12 T) cancer cells were labeled with the proliferation dye carboxyfluorescein succinimidyl ester (CFSE) and cultured in these conditioned media. Notably, cancer cells exposed to the sgIL1RN supernatant retain an average of 30% less of the CFSE signal 4 days after staining (fig. S8D).

In summary, *IL1RN* methylation editing alters macrophage functions in response to inflammatory and pathogenic stimuli, as indicated by changes in cytokine production. The genes encoding the most consistently differentially secreted cytokines, including IL-1RN, IL-1β, and IP10, are highly expressed in ISG and IL1B monocytes and up-regulated in the tumor microenvironment. Therefore, *IL1RN* methylation modulation in these monocyte subsets may influence their cytokine output within the tumor microenvironment.

## DISCUSSION

In this study, we combined the highly efficient and homogeneous reprogramming of B cells into macrophages via ectopic C/EBPα expression with CRISPR-dCas9–mediated DNAm editing to investigate the causal link between DNAm and gene expression. To place these findings in a physiological context, we integrated computational analyses of primary human blood cell datasets, with a focus on myeloid cell fate acquisition. Using this approach, we demonstrate that altering the methylation status of the *IL1RN* promoter region is sufficient to trigger substantial changes in gene expression, leading to distinct myeloid cell fate outcomes and responses to inflammatory signals, which in turn affect the behavior of these cells in both functional and pathological contexts.

During the B-to-macrophage reprogramming process, we observed a loss of DNAm at GREs associated with myeloid genes. These GREs included key TFs such as *KLF4* or *MAFB*, DNAm-related enzymes including *TET2*, and genes associated with inflammation, such as the cytokine-coding genes *IL1B*, *IL1RN*, and *IL6* (Fig. 1E). While the involvement of KLF4 (*42*), MAFB (*43*), and TET2 (*12*, *44*, *45*) in immune-related cell fate decisions has been documented previously, the role of the inflammatory pathways has not been extensively explored. The IL-6 pathway was found to be crucial for establishing proper pluripotency during the conversion of mouse embryonic fibroblasts (*46*, *47*) or murine pre-B cells (*48*, *49*) into induced pluripotent stem cells. However, it was found to be dispensable for myeloid acquisition during the conversion of murine pre-B cells into iMacs (*49*, *50*). Conversely, the IL-1 pathway has not been studied in the context of experimentally induced cell fate conversions. Here, we identified GREs associated with the IL-1 pathway–related genes *IL1B* and *IL1RN,* losing DNAm during human B-to-macrophage reprogramming (Fig. 1E). These genes encode for the agonist and the antagonist of the pathway, respectively (*51*). These IL-1 pathway–related GREs losing DNAm coincide with C/EBPα-binding sites. Notably, its binding potentially triggers their DNA demethylation and associated chromatin activation, finally leading to their increased transcriptional activity (fig. S5, A and B). The observed DNA demethylation and chromatin activation by C/EBPα at these GREs might be mediated by its ability to act as a pioneer TF engaging a broad range of chromatin modifiers (*42*, *52*), including TET2 (*12*). Lastly, these findings align with the extensive C/EBPα occupancy observed at regions undergoing DNA demethylation during the cell fate conversion process (fig. S1G).

The IL-1–related GREs that C/EBPα binds to and activates in the human reprogramming system are also occupied by this factor during the homeostatic differentiation of murine HSPCs to GMPs (fig. S5, E and F). This suggests that C/EBPα could be a critical transcriptional regulator of the IL-1 pathway during physiological myeloid establishment. By reducing the levels of *IL1RN,* we found that in our cellular model of human myeloid cell fate acquisition, there was an abnormal shift in the myeloid cell fate and enhanced phagocytic capacity (Fig. 4, A to G, and fig. S4, E to G). All of these changes are consistent with the imbalanced production of blood cells observed in mice chronically exposed to IL-1β (*35*) or those with an *Il1rn* deficiency (*36*), as well as with our findings in primary cells, which indicate that *IL1RN* depletion impairs human myeloid commitment (Fig. 4, H and I, and fig. S4K). This is also in line with the observation that lower levels of *IL1RN* are associated with reduced survival rates in patients with AML, particularly for the more differentiated subtypes M4-M5 (*36*). On the basis of the strong association we observed between *IL1B* and *IL1RN* expression and the methylation status of their GREs, aberrant GRE methylation at these loci may contribute to the dysregulated activation of the IL-1 pathway observed in patients with AML.

In mature myeloid cells, the IL-1 pathway is crucial in responding to inflammatory stimuli (*29*). Overactivity in this pathway leads to various immune disorders that can be managed by treating patients with a recombinant form of IL-1RN known as anakinra (*53*, *54*). One such immune disorder is the deficiency of the IL-1 receptor antagonist, a rare but severe autoinflammatory disease characterized by mutations in the *IL1RN* gene (*55*). However, in most IL-1–related autoinflammatory conditions, such as cryopyrin-associated periodic syndromes (CAPS), no mutations exist in the *IL1RN* or *IL1B* genes. Rather, hyperactivation of the pathway in CAPS may be caused by abnormal methylation at their gene promoters. This molecular condition is correctable by anti–IL-1β treatment (*56*). Thus, it suggests that inflammatory responses involved in certain pathological conditions might be fine-tuned through the methylation status of the *IL1RN* gene. We have proven this possibility using a DNAm editing tool to induce hypermethylation of the *IL1RN* promoter in a physiologically relevant macrophage cellular model (*57*, *58*, *26*). This epigenetic perturbation led to an aberrant cellular response to a broad range of inflammatory and pathogenic stimuli (Fig. 5H and fig. S8A), mirroring the deficient macrophage functional responses observed in IL-1 pathway–associated immune diseases. Specifically, upon IL-1β stimulation, we observed hyperactivation of the p65/NF-κB pathway in both untreated and 3-hour–treated IL-1β iMacs, along with a deficient IFN pathway activation (Fig. 5, A to F, and fig. S6, A to I). The IL-1 pathway and the IFN pathway are distinct inflammatory responses that counter-regulate each other, playing a crucial role in maintaining innate inflammatory balance in both homeostasis and infection (*59*). The impact of the type I IFN pathway on the IL-1 pathway has been extensively researched (*59*). However, little is known about the reciprocal cross-regulation molecular mechanisms (*59*, *60*). Therefore, our *IL1RN*-depleted macrophages displaying an altered IFN activation in response to IL-1β might be instrumental in providing insight into their interplay.

Our iMacs transcriptionally resemble the ISG monocytes (ISG-Mon, 4) population (Fig. 5G and fig. S6L). Of note, ISG monocytes have been associated with lung pathological conditions. For instance, a positive correlation was detected between the percentage of ISG monocytes in the lungs and the severity of the COVID-19 disease (*40*). Our study identified lung and colon cancer–associated ISG and IL1B monocytes displaying higher levels of proinflammatory genes than their healthy counterparts (fig. S8C), potentially involving them in modulating cancer growth in the tumor microenvironment. Lung and colon cancer cells exhibited altered growth when cultured in proinflammatory conditions (fig. S8D), consistent with inflammation’s known role in modulating cancer development (*61*).

While major drug agencies have already approved a CRISPR-Cas9–based DNA editing treatment (*57*), the development of epigenome editing therapies has been delayed because of initial off-target effects and challenges in accurate delivery into the targeted cells (*58*). Nevertheless, preclinical studies show promise, such as using DNAm editors to reverse disease-associated abnormal DNAm events in neurological conditions (*32*, *62*). Notably, a recent breakthrough in epigenome therapy achieved permanent silencing of a gene involved in cholesterol homeostasis directly in the mouse liver (*63*). Therefore, on the basis of recent technological developments, an epigenome editing therapy to modulate the DNAm levels of the *IL1RN* promoter in human myeloid cells might be a reality in the midterm. Our findings suggest that such a therapy might be instrumental in fine-tuning the IL-1 pathway activity across a variety of human conditions, ranging from autoinflammatory diseases to the regulation of leukemia or solid cancer growth.

## MATERIALS AND METHODS

### Cell lines and cell culture

BLaER cells are derived from a human B-cell precursor leukemia cell line (RCH-ACV) that stably expresses the myeloid TF C/EBPα fused with the estrogen receptor (ER) and labeled with green fluorescent protein (*24*). BlaER cells and subclones were grown in suspension in RPMI 1640, HEPES (GIBCO) supplemented with 10% heat-inactivated fetal bovine serum (FBS GIBCO), 1× penicillin-streptomycin (GIBCO), 1× l-glutamine (GIBCO), and 0,1× β-mercaptoethanol (GIBCO). The culture medium was replaced every 2 to 3 days upon counting in a hemocytometer and using trypan blue exclusion dye to discriminate between live and dead cells. Then, cells were seeded at 2 × 10^5^ cells/ml into an appropriate tissue culture flask.

Human embryonic kidney (HEK) 293 T cells were grown in Dulbecco’s modified Eagle’s medium (DMEM) (+) d-glucose supplemented with 10% heat-inactivated FBS, 1× L-glutamine, and 1× penicillin-streptomycin. For optimal growth, all cell lines were kept in a 5% CO_2_ humidified atmosphere at 37°C. The cells were checked for mycoplasma infection every month and tested negative.

### Transdifferentiation of human B cells into macrophages

To induce transdifferentiation of human leukemic B cells (B cells) into iMacs, 2 × 10^5^ BLaER cells were seeded in a 12-well plate. To activate C/EBPα, 100 nM 17-βestradiol (E2) (Sigma-Aldrich), human IL-3 (10 ng/ml) (PeproTech), and human M-CSF (10 ng/ml) (PeproTech) were added to the medium to favor the conversion.

### Lentiviral production and cellular transduction with the DNAm editing tools

To transduce B cells with the DNAm editing tools, low-passaged HEK-293T cells were seeded for transfection. Lentiviruses were produced in these cells by co-transfecting them with lentiviral plasmids VSV-G, psPAX2, and a transfer vector (Fuw-dCas9-DNMT3A-P2A-tagBFP, Addgene, #84569; Fuw-dCas9-TET1-P2A-tagBFP, Addgene, #108245; and pLV GG hUBC-dsRED, Addgene, #85034) using calcium phosphate. Supernatants containing lentiviral particles were collected and filtered at 48- and 96-hours posttransfection and concentrated by centrifugation at 70.000*g* for 2 hours at 10°C. BLaER cells were then spin-infected (1000*g*; 32°C for 90 min) with the concentrated viruses. Single-cell sorting of the BFP^+^ population was performed to generate stable dCas9-DNMT3A-P2A-BFP (hereafter dCas9-DNMT3A) or dCas9-TET1-P2A-BFP (hereafter dCas9-TET1) clones. Single-sorted cells were then grown at 37°C in 20% FBS-RPMI medium for 14 to 20 days to generate individual clones containing the dCas9-DNMT3A or the dCas9-TET1 epigenome editing tool. To modify the methylation status, dCas9-DNMT3A and dCas9-TET1 clones were selected (based on their dCas9/BFP expression levels). Next, selected clones were infected with lentiviruses harboring simultaneously four sgRNAs targeting the *IL1RN* gene promoter (sgIL1RN) and scramble regions (sgCTRL).

The design of the sgRNAs targeting the *IL1RN* promoter (chr2:113,127,440-113,127,701) and CTRL regions was performed using Benchling sgRNAs design tool for CRISPR (https://benchling.com/crispr). sgRNAs close to a protospacer adjacent motif with 5′-NGG-3′ were selected with the best on-target and off-target scores. Multiplex sgRNA-dsRED (pVL GG hUBC-dsRED plasmid; Addgene, # 84034) construct was cloned using a two-step protocol as described by Engler and co-workers (*64*, *65*). Briefly, each protospacer was first annealed and cloned into the desired expression vector [phH1-gRNA (Addgene, # 53186), ph7SK-gRNA (Addgene, #53189), pmU6-gRNA (Addgene, # 53187), and phU6-gRNA (Addgene, #53188)] using a BbsI restriction enzyme site. Next, four promoter-gRNA cassettes were cloned into the lentiviral destination vector GG hUBC-dsRED using Golden Gate assembly (*66*). Plasmids were sequenced using the M13 Reverse primer. sgRNA sequences are listed in table S2.

### Immunophenotype profiling of methylation edited macrophages

To isolate CD14-positive monocytes for the immuneprofiling, buffy coats were obtained from anonymous donors via the Catalan Blood and Tissue Bank (CBTB). The CBTB follows the principles of the World Medical Association Declaration of Helsinki. Before providing blood samples, all donors received detailed oral and written information and signed a consent form at the CBTB. The use of these samples was approved by the Clinical Research Ethics Committee of Hospital Universitari Germans Trias i Pujol (PI-20-129). Peripheral blood mononuclear cells (PBMCs) were isolated by density-gradient centrifugation using lymphocyte-isolation solution (Rafer, catalog no. L0560-10). Pure monocytes were then isolated from PBMCs by positive selection with magnetic CD14 MicroBeads (Miltenyi Biotech, catalog no. 130-050-201). Monocytes were grown in RPMI 1640 supplemented with 1% penicillin-streptomycin. After 30 min, the monocytes were attached to the cell culture plates. Then, the medium was supplemented with 10% heat-inactivated FBS (GIBCO). To differentiate the monocytes into macrophages, the cells were treated with human M-CSF (10 ng/ml; PeproTech, catalog no. 17862143) or human GM-CSF (10 ng/ml; PeproTech, catalog no. 17851803) during 7 days.

All antibodies used in the staining for the spectral cytometry analysis are listed in table S3. Briefly, one million GM-CSF– or M-CSF–derived monocytes and CTRL- or sgIL1RN-iMacs were washed with phosphate-buffered saline (PBS). Then, the cells were stained with the “viability mix” (table S6) for 15 min at room temperature (RT) in the dark, washed with 3 ml of PBS, and centrifuged. After viability staining, the cells were sequentially stained by incubating with “post-viability mix” for 10 min, “chemokine mix” for 10 min, and “general master mix” for 20 min, protected from light. Then, the cells were washed with PBS twice, resuspended in 300 μl of PBS, and acquired on a Cytek Aurora 5-laser spectral flow cytometer. Spectral cytometry analysis was performed using FlowJo Software for initial gating (viability, SSC-A/FSC-A doublets) and for collecting the mean fluorescence intensity of the different markers.

### Immunocytochemistry and microscopy for p65

To assess p65 cellular localization, dCas9-DNMT3A CTRL and sgIL1RN iMacs were seeded at 0.8 × 10^6^ cells/ml on poly-lysine–coated coverslips. After 2 days, iMacs were treated with IL-1β (1 ng/ml) for 3 hours. Next, the cells were fixed with 4% paraformaldehyde (in PBS) for 20 min and permeabilized with PBS + Triton X-100 0.5% for 10 min. The coverslips were washed twice with PBS, blocked with blocking solution [PBS + bovine serum albumin (BSA) 4% + 0.025% Tween 20] and incubated with an anti-p65 antibody (1:200; Abcam, catalog no. ab16502) overnight at 4°C. After washing, the cells were incubated with an anti-rabbit Alexa Fluor 647 (1:300; Invitrogen, catalog no. A21245) for 1 hour at RT. After four washes with PBS, cells were stained with 4′,6-diamidino-2-phenylindole (DAPI, 2 μg/ml) and mounted into slides using Vectashield (Vector Laboratories, catalog no. H-5700-60). Images were obtained with a Leica TCS-SL confocal microscope. ImageJ was used to quantify the mean intensity of the nucleus/cytoplasm ratio for p65 signal. Twenty cells were counted for each sample.

### Phagocytosis assay

Phagocytosis assays were performed using dCas9-DNMT3A CTRL and sgIL1RN iMacs. The assays were also performed in iMacs containing shRNA (shLuc and shIL1RN_1/2). In both cases, cells were seeded at 0.5 × 10^6^ cells/ml in DMEM. Next, Fluoresbrite carboxyl bright blue beads (1 μm; Polysciences, catalog no. 17458-10) were added to the media (300 beads per cell) and incubated for 24 hours before fluorescence-activated cell sorting (FACS) analysis. Data were analyzed with FlowJo software.

### shRNA-mediated *IL1RN* silencing

For constitutive shRNA-mediated gene silencing, oligonucleotide pairs encoding *IL1RN* shRNAs were annealed and cloned into pSICOR-PGK-puro (Addgene, #12084). pSicoOligomaker 1.5 (https://venturalaboratory.com) was used to select and design oligos. The following shRNAs were used:

*Luciferase* shRNA (shLuc): CCTAAGGTTAAGTCGCCCTCG

*IL1RN* shRNA_1 (sh1): GCGTCATGGTCACCAAATT

*IL1RN* shRNA_2 (sh2): GTACTATGTTAGCCCCATA.

BlaER cells were transduced with shLuc or shIL1RN lentiviruses and selected with puromycin (1 μg/ml) for 2 days.

### Surface markers profiling in shRNA-transduced macrophages

Stainings for CD14 and CD11b were conducted in iMacs with shRNAs (shLuc and shIL1RN_1/2). Briefly, the cells were collected and incubated with human FcR binding inhibitor (eBiosciences, catalog no. 16916173) to avoid unspecific staining. Next, the cells were stained against the macrophage marker CD11b (1:200 of CD11b-APC, BD Pharmingen, catalog no. 550019) and CD14 (1:200 of CD14-APC, BD Pharmigen, catalog no. 561383) and resuspended in PBS containing DAPI as a viability marker.

### Primary human CD34^+^ cell transduction and colony-forming unit assay

Primary human bone-marrow CD34^+^ cells (STEMCELL Technologies, catalog no. 70002.3) were cultured in StemSpan SFEM (STEMCELL Technologies, catalog no. 9600) supplemented with StemSpan TM CC100 (STEMCELL Technologies, catalog no. 2690). shLuc or IL1RN shRNA_1/2–expressing viruses were centrifuged onto RetroNectin-coated plates prepared according to manufacturer’s instructions. Thirty thousand cells were transduced in virus-coated plates by spin infection at 1000*g* for 2 hours at 32°C. The day after, the cells were selected with puromycin (1 μg/ml) for 2 days. To assess colony formation, 1000 cells were plated on MethoCult in biological triplicates (STEMCELL Technologies, catalog no. H4434) according to manufacturer’s instructions. Colonies (BFU-E, CFU-M, CFU-G, CFU-GM, and CFU-GEMM) were counted and scored at day 14 using standard morphological criteria.

To check knock-down efficiency, RNA was extracted from 10,000 CD34^+^ cells with AllPrep DNA/RNA Micro Kit (Qiagen, catalog no. 80284). RNA was converted into cDNA using the RNA to cDNA kit (Applied Biosystems) following the manufacturer’s instructions. Next, cDNA was preamplified using Taqman preamp kit (Thermo Fisher Scientific, catalog no. 384266) following the manufacturer’s instructions. Real-time quantitative polymerase chain reaction (qPCR) reactions were performed using SYBR Green reagent and analyzed using QuantStudio 5 System (Applied Biosystems).

### CSFE proliferation assay in cancer cells

To assess cell proliferation, dCas9-DNMT3A CTRL and sgIL1RN iMacs were treated for 24 hours with IL-1β (1 ng/ml). Then, 2 million HCT-116 or Cal-12t cancer cells were stained with CFSE (Invitrogen, catalog no. C34570) following the manufacturer’s instructions. Next, the cancer cells were seeded at 10,000 cells/ml and grown with the supernatant collected from CTRL or sgIL1RN iMacs. The cells were analyzed for CFSE levels using a FACS Canto II flow cytometer. Data were analyzed with FlowJo software.

### Western blotting

Cells were lysed in Laemmli sample buffer [8% SDS, 40% glycine, 20% β-mercaptoethanol, 0.250 M tris (pH 6.8), and 0.008% bromophenol blue] and boiled for 5 min at 95°C. Protein extracts were separated by electrophoresis and transferred to a nitrocellulose membrane. Membranes were blocked with 5% nonfat milk or 5% BSA for 1 hour at RT. Then, the membranes were incubated overnight at 4°C (while shaking) with the following primary antibodies: goat anti-IL1RN (RD Systems, catalog no. AF280NA) and rabbit anti–cleaved-IL-1β Asp^116^ (Cell Signaling Technology, catalog no. 83186) in 1:1000 in blocking solution and mouse anti-β actin (MERCK, catalog no. A1978) and rabbit anti-vinculin (Cell Signaling Technology, catalog no. 18799) 1:5000 in blocking solution. The membranes were washed three times in tris-buffered saline–Tween before incubation with a secondary antibody anti-mouse immunoglobulin G (IgG) Alexa Fluor 790 nm (Invitrogen, catalog no. A11375), anti-goat IgG Alexa Fluor 800 nm (Invitrogen, catalog no. A32930), or anti-rabbit IgG Alexa Fluor 680 nm (Invitrogen, catalog no. A-21109) 1:5000 in blocking solution for 1 hour at RT. Last, the membranes were developed in an Odyssey CLx system. Image Studio Lite 5.2 (Li-COR) was used for visualization and quantification analysis.

### Quantification of IL-1β by ELISA

Human IL-1 β uncoated enzyme-linked immunosorbent assay (ELISA, Thermo Fisher Scientific, catalog no. 88-7261) was used to quantify IL-1β cytokine in the supernatant of BlaER cells at 0 (B cell), 24, 96, and 168 hours (iMac) of transdifferentiation. Concentrations in (picogram per milliliter) were calculated from a standard curve according to the manufacturer’s instructions.

### Cytokine measurements

The LEGENDplex Human M1/M2 Macrophage Panel (10-plex) (BioLegend, catalog no. 740508) was used to quantify proinflammatory (IL-12p70, TNF-α, IL-6, IL-1β, IL-12p40, IL-23, and IP-10) and anti-inflammatory (IL-10, TARC, and IL-1RA) cytokines in the supernatant of dCas9-DNMT3A CTRL and sgIL1RN iMacs treated for 24 hours with: CpG (3 μM; InvivoGen, catalog no. ODN 2006), poly(I:C) (10 μg/ml; InvivoGen, catalog no. 31852-29-6), human TNF-α (10 ng/ml; PeproTech, catalog no. 300-01A-50UG), and IL-1β (1 ng/ml; PeproTech, catalog no. 200-01B-10UG). The recommended filter plate method was used, and all steps were followed per the manufacturer’s protocol. Samples were acquired in a FACS Canto II flow cytometer, and LEGENDplex Data Analysis Software (BioLegend) was used for data analysis according to the manufacturer’s recommendations. Samples were run in triplicates and included technical duplicates.

### Real-time reverse transcription qPCR

Total RNA was extracted using TRIzol (eBioscences). Five hundred nanograms of total RNA was converted into cDNA using the RNA to cDNA kit (Applied Biosystems) following the manufacturer’s instructions. Real-time qPCR reactions were performed using SYBR Green reagent and analyzed using QuantStudio 5 System (Applied Biosystems). *HPRT and B2M* were used as housekeeping genes. Unpaired Student’s *t* test was used to determine statistical differences in gene expression among the different samples tested (*t* test, ***P < 0.001). Normality and homogeneity in variance were assumed for RT-qPCR experiments with biological triplicates. Primer sequences are listed in table S4.

### RNA-seq

For the transdifferentiation experiment, approximately 1 to 2 × 10^6^ dCas9-DNMT3A CTRL or sgIL1RN cells were collected in duplicates at 0, 3, and 7 days after C/EBPα induction. For the IL-1β treatment experiment, dCas9-DNMT3A CTRL or sgIL1RN iMacs were collected in duplicates at 0, 3, and 16 hours after IL-1β (1 ng/ml) treatment. Total RNA was extracted with the RNeasy Mini Kit (QIAGEN, catalog no. 74104) according to the manufacturer’s instructions and quantified with NanoDrop spectrophotometer. RNA (0.2 to 0.5 μg) was used for mRNA sequencing with poly-A enrichment. Briefly, quality control with fragment analyzer (Agilent) was performed before library preparation to ensure proper RNA integrity (RIN > 7). A DNABSEQ eukaryotic strand-specific mRNA library protocol was used for library preparation. Then, libraries were sequenced in a DNABSEQ-G400 sequencer using a pair-end 150-bp protocol. More than 35 million reads were obtained for each sequenced sample.

### Bisulfite conversion and pyrosequencing

Sample DNAm status was assessed by bisulfite (BS) pyrosequencing. Briefly, 1 μg of genomic DNA was BS-converted using the EZ DNA Methylation Gold Kit (Zymo Research, catalog no. D5006) following the manufacturer’s instructions. BS-treated DNA was PCR-amplified using the IMMOLASE DNA polymerase Kit (Bioline). Primers used for the PCR were designed with PyroMark Assay Design 2.0 software (QIAGEN) and listed in table S5. PCR products were pyrosequenced with the Pyromark Q48 system (QIAGEN) according to the manufacturer’s protocol and analyzed with PyroMark Q48 Autoprep (QIAGEN).

### Whole-genome bisulfite sequencing

Genomic DNA was extracted from 1 million cells at 0, 24, 96, and 168 hours of transdifferentiation using the DNeasy Blood & Tissue kit (QIAGEN, catalog no. 69504) following the manufacturer’s instructions and quantified using Qubit dsDNA (Invitrogen, catalog no. Q32851). Cytosine conversion, library preparation and sequencing were done by the provider of the sequencing services. Briefly, genomic DNA was fragmented to 200 to 400 bp and BS converted. For library construction, sequencing adapters were ligated, followed by double-strand DNA synthesis and PCR amplification. Next, the libraries were sequenced on Illumina HiSeqTM2500 using a pair-end 150-bp protocol rendering >70 Gb per sample. Raw data quality assessment was performed, and low-quality reads were removed.

### DNA methylation arrays

To assess potential off-target DNAm events caused by the dCas9-DNMT3A tool, we used the Infinium MethylationEPIC v2.0 Bead-Chip arrays (Illumina, catalog no. 20087706). This platform allows the interrogation of around 935,000 CpG sites per sample at single-nucleotide resolution, covering 99% of the reference sequence (RefSeq) genes. One million CTRL or sgIL1RN cells were collected at 0 and 3 days after C/EBPα induction, with four biological replicates for each group. Genomic DNA was extracted, BS-converted, and used to hybridize the methylation arrays following the manufacturer’s instructions. Raw files (IDAT files) were provided by the Genomics Unit of the Josep Carreras Research Institute (Barcelona).

### Chromatin immunoprecipitation

ChIP experiments were performed as previously described (*12*). Briefly, 30 million cells were cross-linked with 1% formaldehyde in RPMI 1640 medium while rotating for 10 min at RT. To stop fixation, glycine was added to a final concentration of 0.125 M and rotated for 5 min. Next, the collected cells were washed twice with ice-cold PBS and resuspended in cold immunoprecipitation (IP) buffer {1 volume SDS buffer [100 mM NaCl, 50 mM (pH 8.1), tris-HCl, 5 mM (pH 8) EDTA, 0.2% NaN3, and 0.5% SDS]} and 0.5 volume Triton X-100 dilution buffer [100 mM NaCl, 100 mM (pH 8.6) tris-HCl, 5 mM (pH 8) EDTA, 0.2% NaN3, and 5% Triton X-100] supplemented with proteinase inhibitors (Roche, catalog no. 118733580001). Chromatin was sheared to 100- to 300-bp fragments on a Bioruptor pico sonicator (Diagenode) at 4°C for 13 cycles with 30-s on and 30-s off in 15-ml Bioruptor Tubes (Diagenode, catalog no. C30010017) with 650 mg of beads (Diagenode, catalog no. C03070001). After sonication, cell lysate was spun down at 20,000*g* for 20 min at 4°C to remove debris and 5% of supernatant was saved as input. Chromatin was conjugated with 10 μg of anti-Cas9 antibody (Active Motif, catalog no.61757) while rotating ON at 4°C. The next day, 50 μl of Dynabead A/G mix were blocked for 2 hours at 4°C with BSA (5 mg/ml) while rotating and added into antibody-cell lysate mixture for IP for 3 hours at 4°C in rotation. Chromatin-antibody-bead complexes were washed three times with ice-cold low-salt buffer [50 mM (pH 7.5) Hepes, 140 mM NaCl, and 1% Triton X-100] and once with ice-cold high-salt buffer [50 mM (pH 7.5) Hepes, 500 mM NaCl, and 1% Triton X-100]. Bound protein-DNA complexes were de-cross-linked in elution buffer (1% SDS and 0.1 M NaHCO_3_) by overnight incubation at 65°C with shaking at 1300 rpm. The next day, the eluted portion was treated with ribonuclease for 1 hour at 37°C and then with Protein K for 2 hours at 65°C. Last, DNA was purified by phenol:chloroform:isoamyl alcohol (25:24:1) extraction and ethanol precipitated.

For dCas9-TET1 ChIP-qPCR analysis, DNA was diluted 1:10, and relative enrichment was calculated as a percentage of input with the following formula [100*2^(Adjusted input − Ct (IP)]. Oligonucleotide sequences are indicated in table S6.

For dCas9-DNMT3A ChIP-seq, the samples were quantified with Agilent 2100 before library preparation. Library preparation and sequencing were performed by the sequencing service provider using a DNABSEQ-G400 sequencer and a SE50 protocol.

### Bioinformatic analyses

All sequencing data obtained were mapped onto the human genome assembly hg38 (Ensembl GRCh38) and analyzed with R (4.2.1) using packages from the Bioconductor suite (v3.0) (*67*). For peak calling, regions overlapping the “Encode blacklist” regions were removed (*68*), as well as mitochondrial reads. Peaks were annotated to genomic features in R with the package ChIPseeker (v1.32.1) (*69*) using Benjamini-Hochberg FDR corrections. All GO enrichment analyses were performed using the clusterProfiler package (v4.4.4) (*70*). Pathway enrichment analysis was performed with the Reactome Pathway Database Analysis Tool (*71*). GSEA was done using MSigDB gene sets (*72*), and analyses were performed in R with the fgsea (v1.30.0) (*73*) and clusterProfiler packages (v4.4.4). Bigwig tracks were generated using DeepTools BamCoverage (3.3.1) (*74*). Motif enrichment analyses were performed using the function findMotifsGenome.pl from the package HOMER (v0.2) (*75*). The integration of DNAm and ATAC-seq data was carried out using the bedtools (v2.31.1) (*76*) package. A curated collection of TF targets was obtained from the Collection of Transcriptional Regulatory Interactions (*77*) with the package decoupleR (v2.12.0) (*78*). Both positive and negative interactions were considered for the downstream analysis. Heatmaps and clustering analyses were performed using the ComplexHeatmap (v2.10.0) package (*79*). The Multidimensional Scaling and principal components analysis were performed using the limma (v3.52.2) (*80*), and factoextra packages (v1.0.7), respectively. Upset plots were generated using the upsetR package (v1.4.0) (*81*). The correlation analyses were performed and plotted with the R package corrplot (v0.92). The RNA-seq MA plots were generated using the R package DESeq2 (v1.36.0) (*82*). The remaining plots were generated using the R package ggplot2 (v3.4.2). Genome-wide statistical analyses were performed using a two-tailed Student’s *t* test to compare two groups using the R package stats (v4.2.1).

### WGBS analysis during transdifferentiation

Raw sequence reads from WBGS libraries were trimmed to remove poor-quality reads and adapter contamination using the package Trimmomatic (v0.36) (*83*). The remaining sequences were mapped using Bismark (v0.16.3) (*84*) to the human reference genome GRCh38 in paired-end mode. Reads were then deduplicated, and CpG methylation calls were extracted from the deduplicated mapping output using the Bismark methylation extractor in paired-end mode. The estimation of DNAm levels was calculated by dividing the count of reads identifying a cytosine (C) by the sum of reads identifying either a cytosine (C) or a thymine (T). Only CpGs with coverage equal to or higher than 5× in all tested samples were used for downstream analyses. *N* = 24,298,869 CpGs were analyzed. The genome was split into 1-Kb bins using the function makewindows from the bedtools (*76*) package. DNAm signal was quantified by calculating the mean methylation on these 1-Kb bin regions. Only bins with at least three CpGs covered at least 5× in all tested samples were used for downstream analyses. *N* = 2,807,124 bins were analyzed. Dynamic DNAm bins containing GREs were identified by overlapping those bins with ATAC-seq peaks, either located at a TSS (promoter) or at an H3K4me1 peak, as previously described (*26*).

### WGBS and expression analyses in human primary cells

Publicly available datasets from human primary blood cells were obtained from the Blueprint Project repository (https://projects.ensembl.org/blueprint/). Average DNAm quantification in regions of interest was obtained from the processed BigWig files from the WGBS datasets following a threshold of at least X3 coverage for each CpG. Average DNAm difference of at least 10% between the groups was considered for the downstream analyses. DEGs were detected from RNA-seq data using the processed RSEM gene-level estimated counts using the R package DESeq2 (v1.36.0) following the tximport() pipeline. All DEGs with an FDR < 0.05 between the groups were considered for the downstream analyses.

IL1RN expression during normal hematopoiesis was obtained from the BloodSpot 3.0 database (https://bloodspot.eu/). The “Normal human hematopoiesis (DMAP)” dataset was used to obtain mRNA expression levels of microarray data (log_2_) selecting for the *IL1RN* probe with the overall highest intensity.

### Analysis of DNA methylation arrays

IDAT raw data from the MethylationEPIC BeadChip 850 k v2.0 microarrays were loaded using R to perform all the analyses, QC, and preprocessing steps using the package minfi (v1.42.0) (*85*). Probes with low-detection *P* value (<0.1), probes with a known single-nucleotide polymorphism at the CpG site, and known cross-reactive probes were removed. For the resulting CpGs, beta values were calculated using minfi functions. Differentially methylated positions (DMPs) were calculated using the limma package (v3.52.2) (*80*) in R, adjusting by the Benjamini-Hochberg method. Only DMPs with adjusted *P* values (FDR < 0.05) and a differential of DNAm equal to or greater than 30% (Δβ ≥ 0.3) were selected for further analyses.

### RNA-seq analysis

Reads were mapped using STAR (v2.7.6) (*86*). Gene expression was quantified using the function featureCounts from the package Subread (v2.0.3) (*87*). DEGs were detected using the R package DESeq2 (v1.36.0) (*82*), applying FDR < 0.05. TFs’ activities were inferred from expression values using DoRothEA (*39*).

### scRNA-seq analysis

The processed scRNA-seq dataset of monocytes and macrophages (MoMac-VERSE) is publicly available at the FG Lab Resources website (https://gustaveroussy.github.io/FG-Lab/). Information about cross-tissue integration and data processing was described by Mulder and co-workers (*40*). Data analysis was done in R with the Seurat package (v5.1.0) (*88*). Preestablished clustering counts normalization/scaling and metadata for paired healthy versus cancer conditions were used for the downstream analysis. For gene signature enrichment (up-regulated versus down-regulated) analysis, only the most significantly DEGs (log_2_FC > 0.5, FDR < 0.05) at each time point were considered. For statistical analysis, healthy versus cancer comparisons were evaluated for cell populations that presented at least 15 cells for each condition. Significant differences in gene expression were calculated using two-sided Wilcoxon rank-sum tests with the compare_means() function in R ggpubr package (v0.6.0).

### ChIP-seq analysis

For the ChIP-seq analysis, reads were trimmed using the TrimGalore package (v0.6.6) to remove adaptors and mapped using Bowtie2 (v2.4.4.1) (*89*). Duplicated reads were removed with the tool MarkDuplicates from the package Picard (v3.1.0). Peaks were called using MACS2 (v2.2.5), parameter (-q 0.05). Differential enrichment analysis was performed using the edgeR (*90*) function from the diffBind package (v3.6.5) and filtering by FDR < 0.05 and log_2_FC > 2.

## Supplementary Materials

### This PDF file includes:

Figs. S1 to S8 Tables S1 to S6 References
