# Supplementary material for: Modulating immune cell fate and inflammation through CRISPR-mediated DNA methylation editing
Source: Sci Adv. 2025 Jul 16;11(29):eadt1644. doi: 10.1126/sciadv.adt1644 (PMC12266127; doi:10.1126/sciadv.adt1644)
Supplement: Supplementary file 1 — Figs. S1 to S8 Tables S1 to S6 References [file sciadv.adt1644_sm.pdf]

Science Advances
AAAS

Supplementary Materials for

Modulating immune cell fate and inflammation through CRISPR-mediated DNA methylation editing

Gemma Valcárcel *et al.*

Corresponding author: José Luis Sardina, jsardina@carrerasresearch.org

Sci. Adv. **11**, eadt1644 (2025)
DOI: 10.1126/sciadv.adt1644

This PDF file includes:

Figs. S1 to S8
Tables S1 to S6
References

SUPPLEMENTARY FIGURES

Valcárcel_Supp. Fig 1

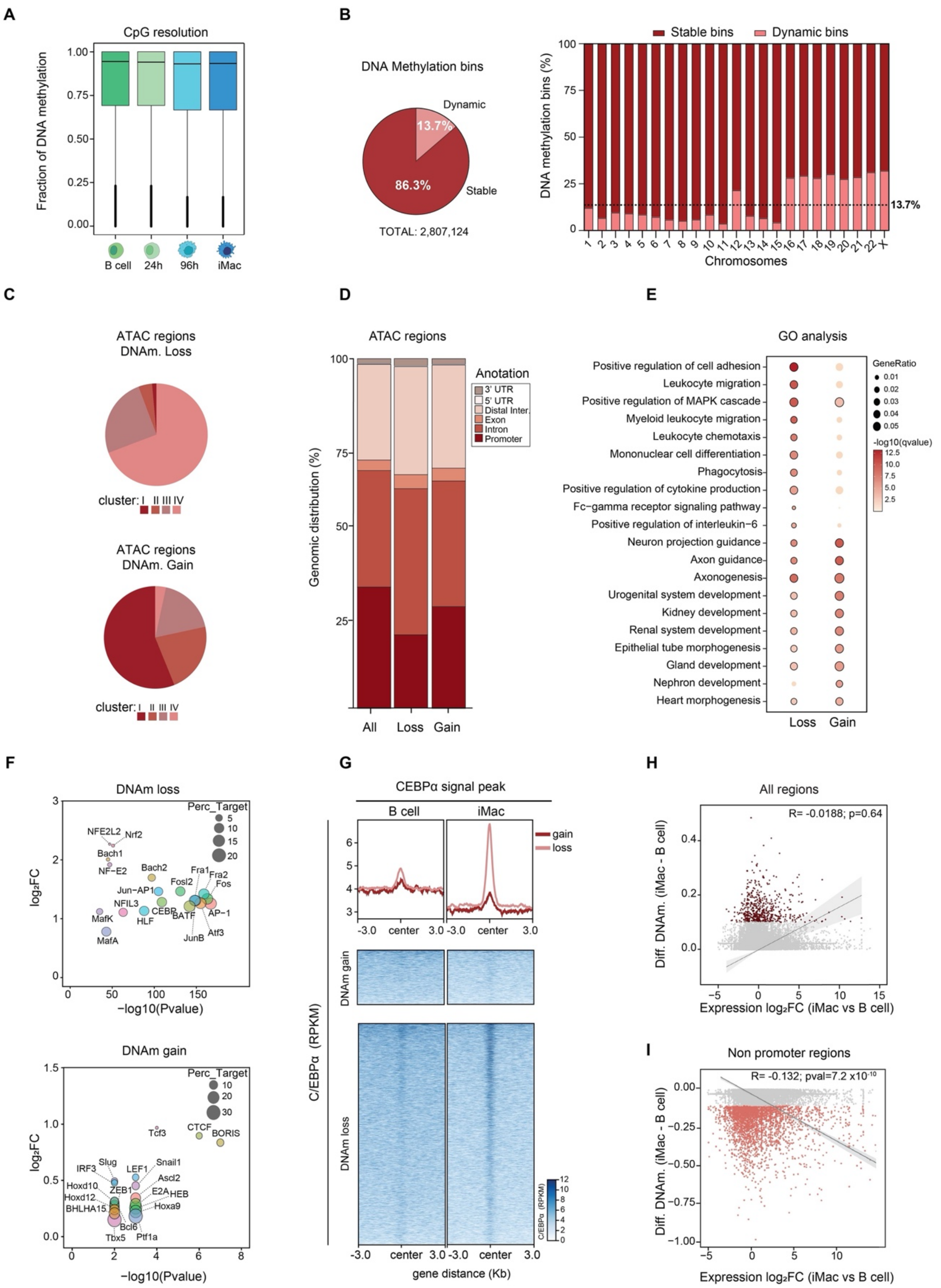

Fig. S1. Related to Fig. 1.

(A) Genome-wide DNAm levels at CpG resolution during transdifferentiation. Plots display the median (bar), interquartile range (box), and the 95% confidence interval (vertical bar). N (number of CpGs analyzed) = 24,298,869 **(B)** Left: Pie chart showing the genome-wide percentage of DNAm stable and dynamic 1kb-bins observed during

transdifferentiation. N (number of bins analyzed) = 2,807,124; Right: Percentage of DNAm stable and dynamic 1kb-bins observed during transdifferentiation across chromosomes. The dashed line represents the genome-wide percentage of dynamic bins (13.7%). **(C)** Piecharts depicting the proportion of ATAC+ DNAm loss and gain regions in Fig. 1E belonging to the methylation clusters in Fig. 1C. **(D)** Distribution of ATAC+ DNAm loss and gain regions in iMacs along the different genomic features analyzed (promoters, introns, exons, distal intergenic, 5' UTR and 3'UTR regions). All ATAC+ regions analyzed (All) are shown as a distribution control. **(E)** Balloon plot depicting the Gene Ontology (GO) enrichment analysis for the genes associated with the ATAC+ DNAm loss and gain regions in iMacs (related to Fig. 1E). 10 highly significant over-represented biological processes (BP) terms for each category are plotted. Ratio of genes of interest over all unique genes (GeneRatio) and -log10(qvalue) are shown. Significant terms (qvalue<0.05) are highlighted with a black stroke. **(F)** Bubble plot showing the output of transcription factor enrichment analyses (by HOMER2) at ATAC+ DNAm loss (top) or gain (bottom) regions in iMacs. The X-axis reports the –log10(P-value), and the Y-axis depicts the log2FC. The circle size is related to the percentage of target sequences containing the motif. **(G)** Genomic heatmaps and average plots showing C/EBPα ChIP-seq signal (in RPKMs) at the ATAC+ DNAm gain and loss regions (in Fig.1E) in B cells and induced macrophages. **(H)** Plot showing the correlation between the step changes (iMac-Bcells) in DNAm and gene expression at iMac's chromatin accessible regions (ATAC+ peaks). Grey dots: ATAC+ regions not gaining at least 10% in DNAm. Dark red dots: ATAC+ regions gaining at least 10% in DNAm in iMacs. **(I)** Plot showing the correlation between the step changes (iMac-Bcells) in DNAm and gene expression at iMac's chromatin accessible regions (ATAC+ peaks) non-overlapping annotated promoters. Grey dots: Regions not losing at least 10% in DNAm. Light red dots: Regions losing at least 10% of DNAm.

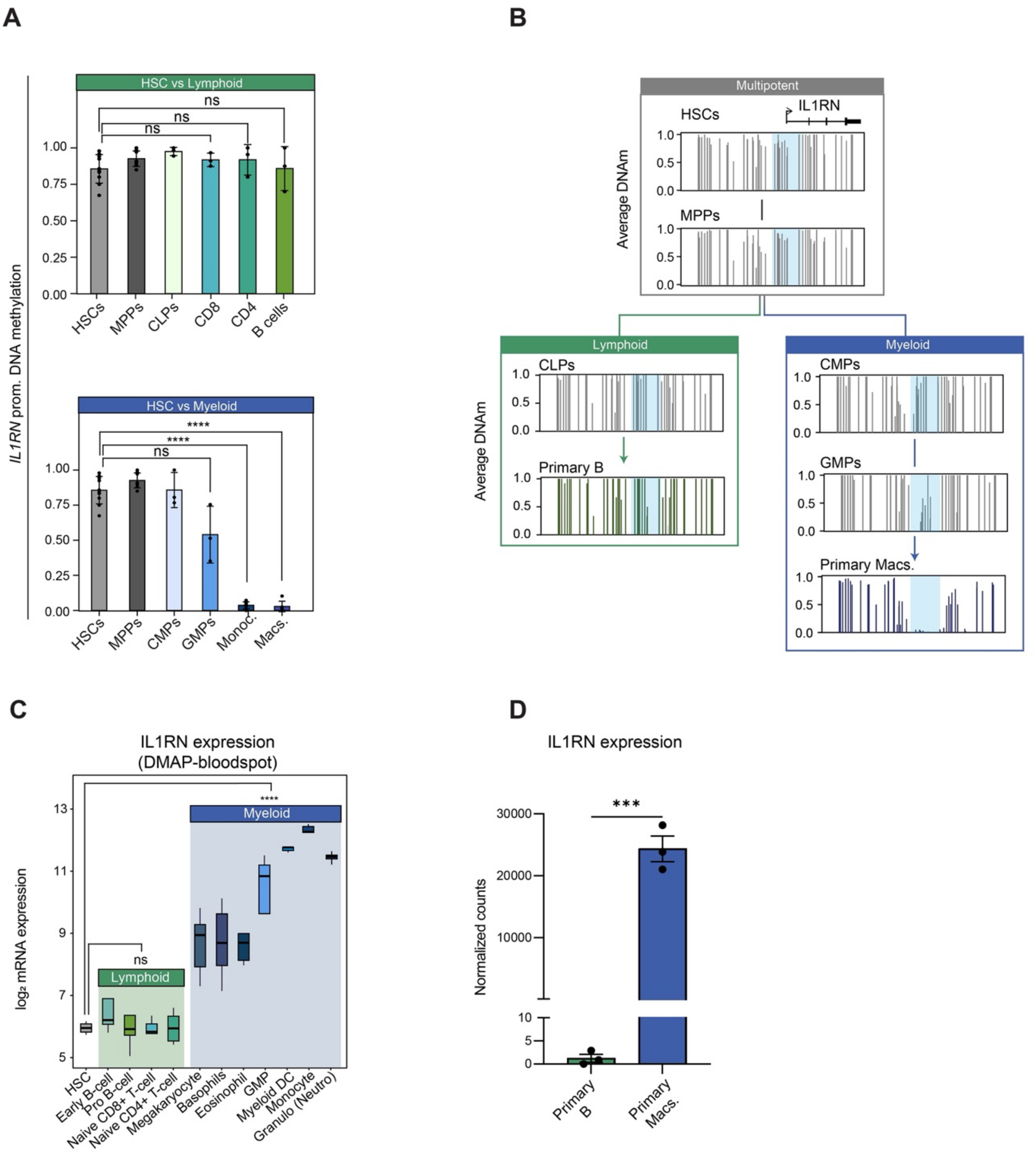

Fig. S2. Related to Fig. 2.

(A) Plots showing the quantification of the DNAm levels at the *IL1RN* promoter in different human primary blood cells. Top: *IL1RN* DNAm levels in HSC vs lymphoid cells. Bottom: *IL1RN* DNAm levels in HSC vs myeloid cells. Unpaired two-tailed Student's t-test, HSCs n=10, MPP n=8, CLP n=3, CD4 n=3, CD8 n=3, B cell n=3, CMP n=3, GMP n=3, Monocytes n=9 and Macrophages n=6; mean ± s.e.m., (****p<0.0001). Data collected from the Blueprint Consortium. **(B)** Genome browser snapshots (chr2: 113125667-113129119) showing signal for DNA methylation (by WGBS) at the *IL1RN* locus across human primary blood cells. The blue-shaded region represents the demethylated area during transdifferentiation (Fig. 1E-G), corresponding to the promoter of the *IL1RN* short isoform (ENST00000409930.4). Data collected from the Blueprint Consortium. **(C)** *IL1RN* mRNA levels (by expression arrays) across human primary blood cells. Unpaired two-tailed Student's t-test, HSCs n=3, Early B n=4, Pro B n=5, naïve CD8 n=7, naïve CD4 n=7, MEP n=), MK n=7, Basophils n=6, Eosinophils n=6, Monocytes n=5, Myeloid DCs n=5, GMPs n=4 and Neutrophils n=4., mean ± s.e.m., (****p<0.0001).

Data collected from Bloodspot (http://www.bloodspot.eu/). **(D)** *IL1RN* mRNA levels (by RNA-seq) in human primary B cells and macrophages. Unpaired two-tailed Student's t-test, n=3 biologically independent samples per group, mean ± s.e.m., (***p<0.001).

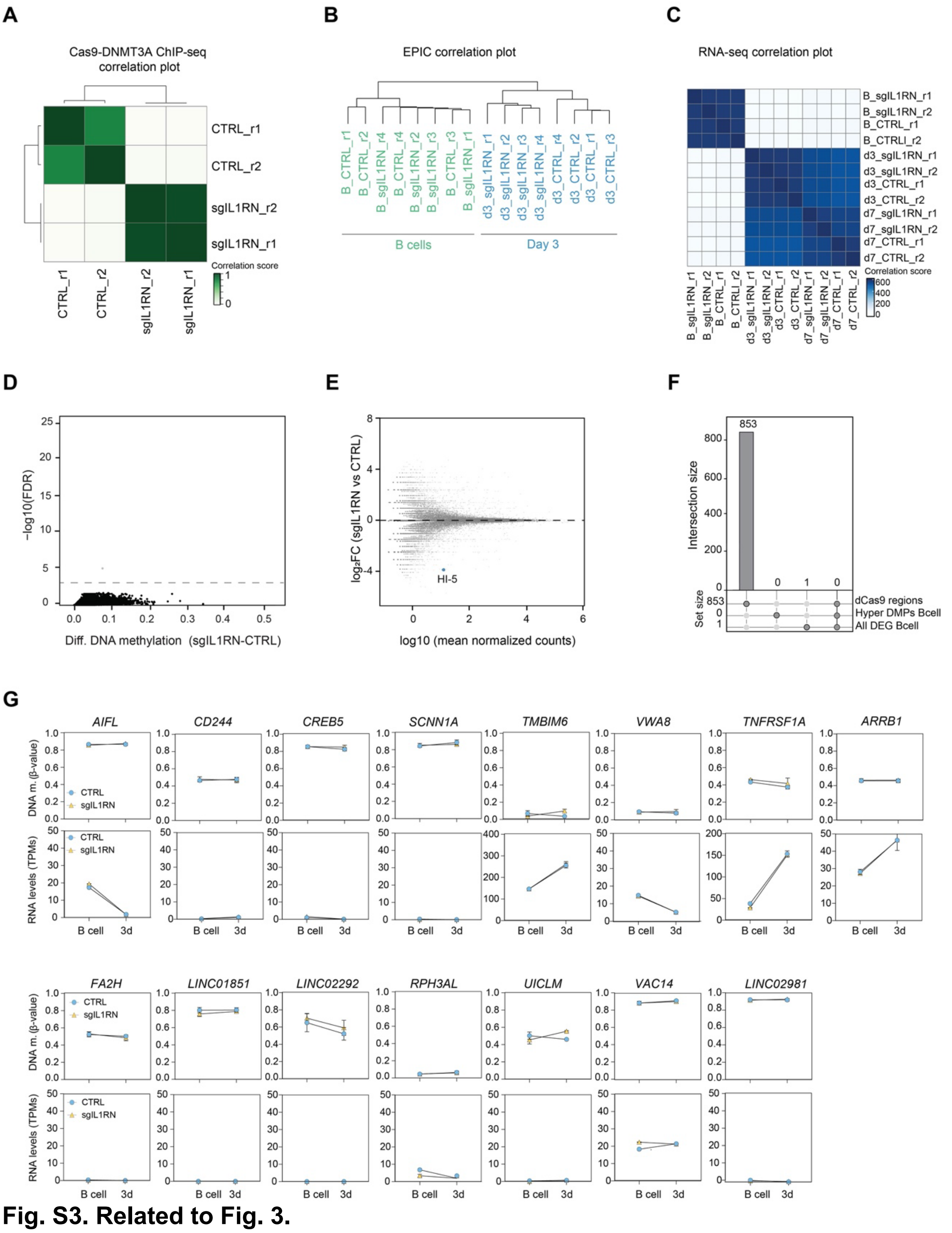

Fig. S3. Related to Fig. 3.

(A) Correlation heatmap showing the correlation (r) values for the Cas9 ChIP-seq experiments in dCas9-DNMT3A CTRL and sgIL1RN B cells. **(B)** Unsupervised clustering of the transdifferentiation samples subjected to the MethylationEPIC BeadChip 850k v2.0 microarrays. **(C)** Correlation heatmap showing the correlation (r) values between the RNA-seq samples during transdifferentiation. Scale bar represents the range of the correlation coefficients (r) displayed. **(D)** Scatter plot showing differentially hypermethylated CpGs in dCas9-DNMT3A sgIL1RN B cells compared to CTRL B cells. Black dots indicate non-significantly hypermethylated CpG positions (FDR>0.05). The grey dot indicates a significantly hypermethylated position (FDR<0.05). No significantly

hypermethylated CpG positions gaining ≥30% of DNAm were observed ($\Delta\beta<0.3$, FDR<0.05, n=0). The dashed line indicates the FDR<0.05. **(E)** MA plot showing differentially expressed genes (DEGs) in dCas9-DNMT3A sgIL1RN B cells compared to CTRL B cells. The blue dot indicates a significant DEGs (FDR<0.05, n=1). **(F)** Upset plot depicting the intersection of the significantly dCas9-DNMT3A bound regions in (Fig. 3G), the significantly hypermethylated $\Delta\beta\geq0.3$ CpG positions in (D), and the significant associated DEGs in (E) in dCas9-DNMT3A sgIL1RN compared to CTRL B cells. No candidate intersects the 3 datasets. **(G)** Plots showing the DNAm (top panels) and transcriptional (bottom panels) dynamics of top dCas9-DNMT3A bound regions (FC>3.5, p<0.05) identified in Fig. 3G. Unpaired two-tailed Student's t-test, n=4 (DNAm) n=2 (RNA-seq) per group, mean ± s.e.m, not significant (p>0.05). Infinium MethylationEPIC BeadChip 850k v2.0 probes used to calculate DNAm levels for each plot are depicted in **Supplemental Table S1**.

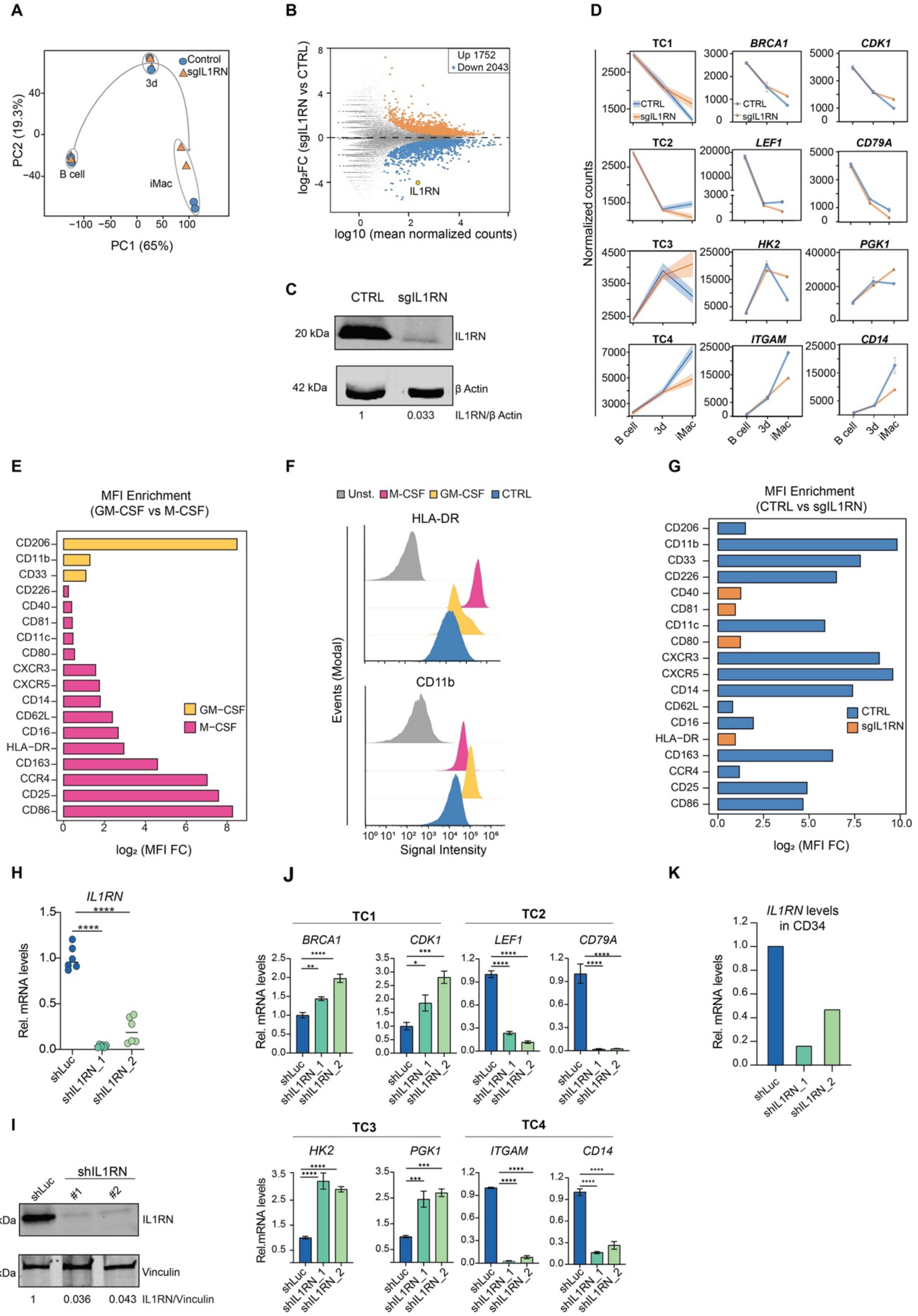

Fig. S4. Related to Fig. 4.

(A) Principal Component Analysis (PCA) displaying the transcriptomic dynamics of dCas9-DNMT3A sgIL1RN and CTRL cells during transdifferentiation. **(B)** MA plot showing differentially expressed genes (DEGs) in dCas9-DNMT3A sgIL1RN iMacs

compared to dCas9-DNMT3A CTRL iMacs. Orange dots indicate upregulated genes (FDR<0.05 n=1,752). Blue dots and the yellow dot (*IL1RN*) indicate downregulated genes (FDR<0.05, n=2,043). **(C)** Representative western blot image of IL1RN protein in dCas9-DNMT3A sgIL1RN and CTRL iMacs. IL1RN levels were normalized to β-Actin levels and expressed as a fold change over CTRL iMacs. **(D)** Left panel: Quantification of RNA signal at the transdifferentiation clusters in (Fig. 4A) for sgIL1RN and CTRL cells; Center and right panels: expression dynamics of representative genes from the clusters. **(E)** Barplot displaying the enrichment ratio of Mean Fluorescence Intensity (MFI) of key macrophage surface markers in PB-monocyte-derived macrophages generated either in the presence of GM-CSF or M-CSF. Yellow bars: positive enrichment MFI ratio in GM-CSF macrophages; Magenta bars: positive enrichment MFI ratio in M-CSF macrophages. **(F)** Representative histograms showing signal intensity for selected cell surface markers (HLA-DR-APC-Fire750 and CD11b-APC) in GM-CSF- or M-CSF-macrophages and in CTRL-iMacs. **(G)** Barplot displaying the enrichment ratio of Mean Fluorescence Intensity (MFI) of key macrophage surface markers in CTRL vs sgIL1RN iMacs. Blue bars: positive enrichment MFI ratio in CTRL-iMacs; Orange bars: positive enrichment MFI ratio in sgIL1RN iMacs **(H)** RT-qPCR analysis of *IL1RN* expression in iMacs harboring shRNAs targeting the luciferase gene (shLuc, control) or the *IL1RN* gene (shIL1RN). Values were normalized against *B2M* expression. Two-way ANOVA with Dunnett's post-hoc test, n=6, mean ± s.e.m., (****p<0.0001). **(I)** Representative western blot image of IL1RN protein in shIL1RN and shLuc iMacs. IL1RN levels were normalized to vinculin protein levels and expressed as a fold change over shLuc iMacs. **(J)** RT-qPCR analysis in shIL1RN and shLuc iMacs of selected genes from the transdifferentation clusters shown in (Fig. 4A and fig. S4D). Values were normalized against *B2M* expression. Two-way ANOVA with Dunnett's post-hoc test, n=3, mean ± s.e.m., (*p<0.05; **p<0.01; ***p<0.001; ****p<0.0001). **(K)** Representative RT-qPCR analysis of *IL1RN* expression in bone-marrow purified human CD34+ cells harboring shRNAs targeting the luciferase or the *IL1RN* gene. Values were normalized against *HPRT* expression.

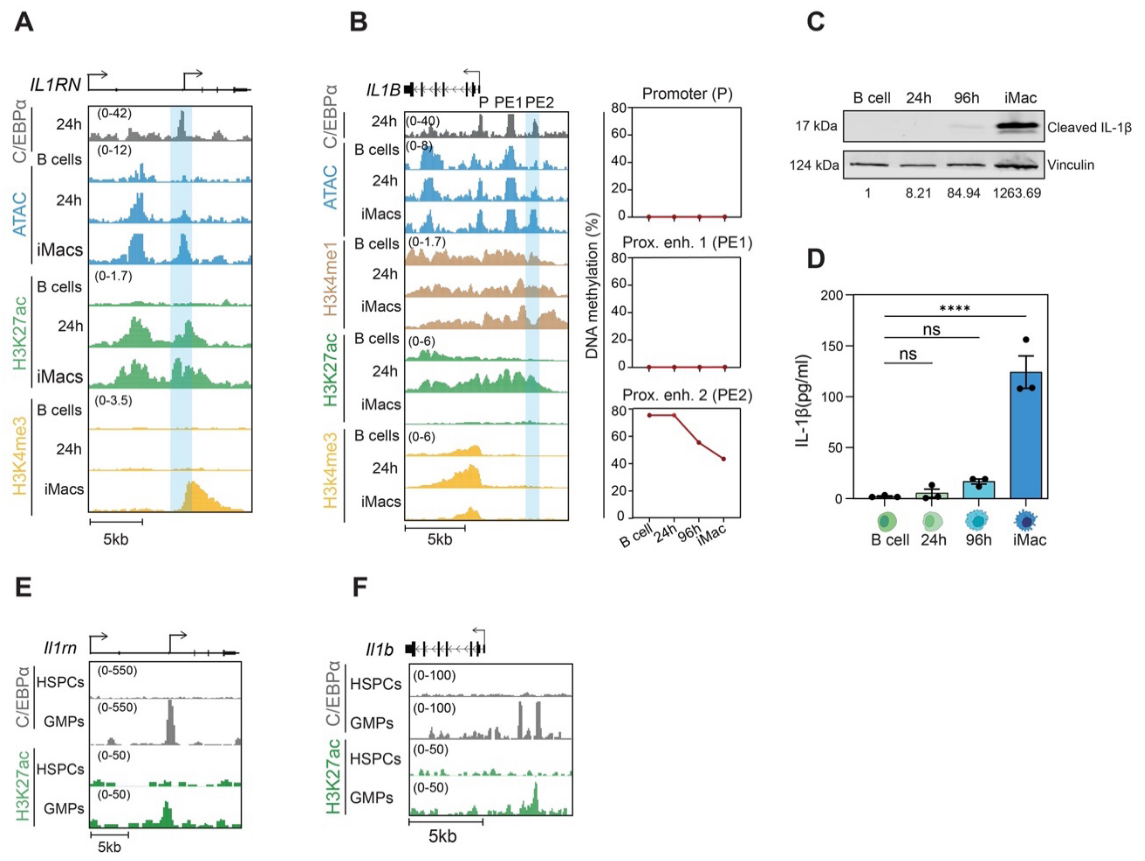

Fig. S5. Related to Fig. 4.
(A) Genome browser snapshot at the human *IL1RN* locus showing signal for C/EBPα ChIP-seq at 24h and chromatin accessibility (by ATAC-seq), H3K27ac and H3K4me3 ChIP-seq signal in B cells, 24 hours, and in iMacs. The blue-shaded region indicates the DNA demethylated region at the *IL1RN* promoter (as in Fig. 1G). **(B)** Left: Genome browser snapshot at the human *IL1B* locus showing signal for C/EBPα ChIP-seq at 24h and chromatin accessibility (by ATAC-seq), H3K4me1, H3K27ac and H3K4me3 ChIP-seq signals in B cells, 24 hours, and in iMacs. At 24h, C/EBPα binds 3 GREs within the *IL1B* locus: the promoter (P) and 2 proximal enhancers (PE1-2). The blue-shaded highlight indicates a DNA demethylation event at PE2 (as identified in Fig. 1E). Right: Quantification of the DNAm kinetics at the 3 IL1B-GREs identified in (B). **(C)** Representative western blot image illustrating the levels of cleaved IL-1β protein during B cell to macrophage transdifferentiation. IL-1β levels were normalized to vinculin levels and expressed as a fold change over B cells. **(D)** Quantification of IL-1β levels in the cellular supernatant (by ELISA) during B cell to macrophage transdifferentiation. One-way ANOVA with Dunnett's post-hoc correction, n=3, mean ± s.e.m., ($p<0.0001$). **(E-F)** Genome browser snapshots at the mouse *Il1rn* locus (F) and the *Il1b* locus (G) showing signals for C/EBPα and H3K27ac ChIP-seq in murine Hematopoietic Stem Progenitor cells (HSPCs) and Granulo-Monocyte Progenitors (GMPs). Data were taken from: H3K27ac and H3K4me3 ChIP-seq during human transdifferentiation (ArrayExpress: E-MTAB-9825); C/EBPα ChIP-seq in murine HSPCs and GMPs (GEO: GSE43007) (*91*) H3K27ac ChIP-seq signal in murine HSPCs and GMPs (GEO: GSE59636) (*92*).

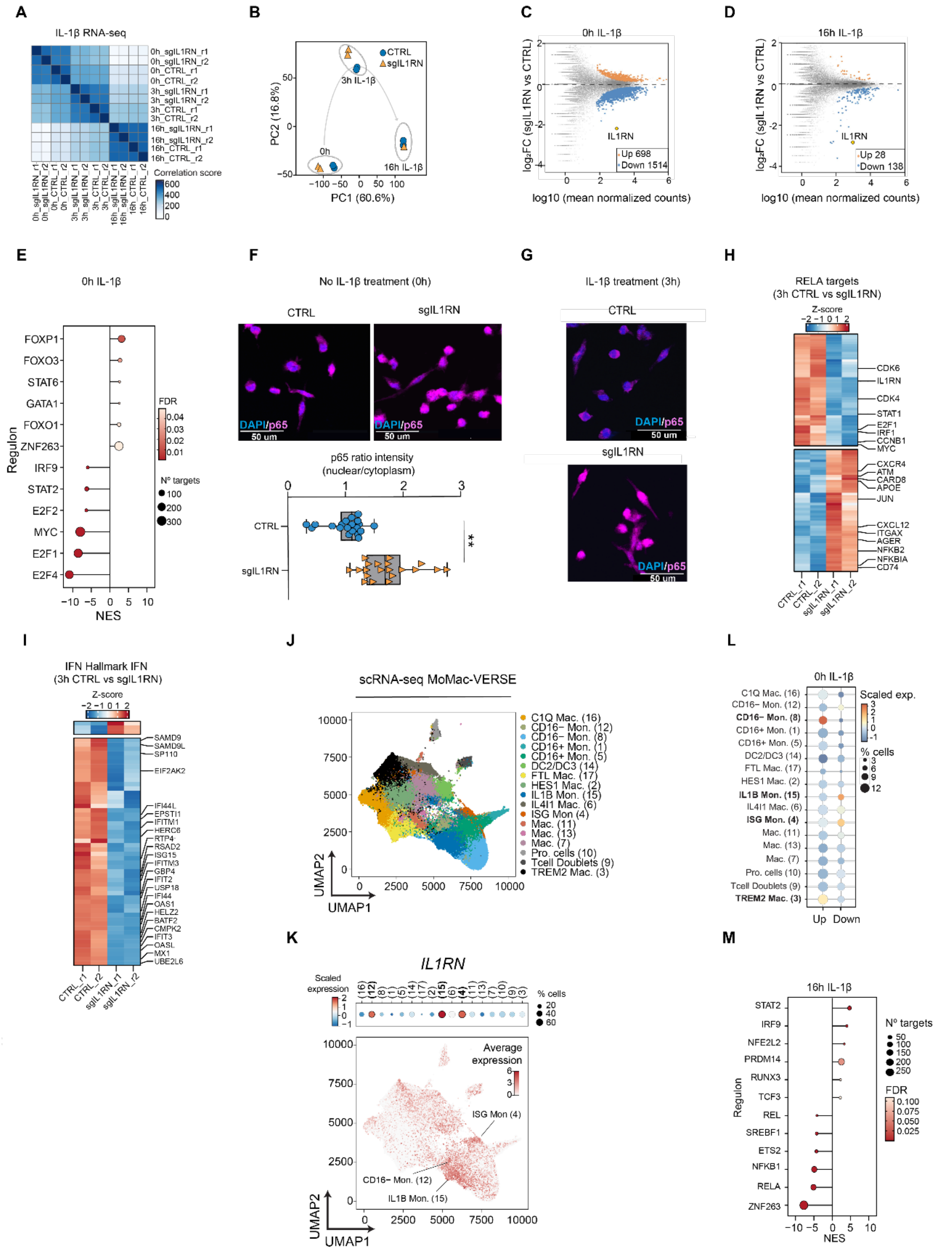

Fig. S6. Related to Fig. 5.
(A) Correlation heatmap showing the correlation (r) values between the RNA-seq samples during IL-1β treatment. Scale bar represents the range of the correlation coefficients (r) displayed. **(B)** Principal Component Analysis (PCA) displaying the transcriptomic dynamics of sgIL1RN and CTRL iMacs treated with IL-1β. **(C-D)** MA plots showing differentially expressed genes (DEGs) in untreated **(C)** and 16h IL-1β-treated

conditions **(D)** dCas9-DNMT3A sgIL1RN iMacs compared to CTRL iMacs. Orange dots indicate upregulated genes (0h=698; 16h=28; FDR<0.05). Blue dots and the yellow dot (*IL1RN*) indicate downregulated genes (0h=1514; 16h=138; FDR<0.05). **(E)** Lollipop plot depicting the TF activity predicted from mRNA expression of target genes with DoRothEA v2.0 (*39*) in untreated (0h) sgIL1RN and CTRL iMacs. Lollipop size indicates the total number of genes regulated by each transcriptional regulon. **(F)** Top: representative IFs showing p65 (red) and DAPI (blue) signals in sgIL1RN and CTRL untreated (0h) iMacs. Bottom: quantification of p65 nuclear versus cytoplasmic localization signal in sgIL1RN and CTRL iMacs. Unpaired two-tailed Student's t-test, n=20 cells per group, mean s.e.m., (**p<0.01). **(G)** Representative Immunofluorescences (IFs) showing p65 (red) and DAPI (blue) signals in sgIL1RN and CTRL iMacs treated with IL-1β for 3h. Quantification shown in Fig. 5D. **(H)** Heatmap showing the expression levels (by RNA-seq) of p65 target genes identified as differentially expressed at 3 hours of IL-1β treatment between CTRL and sgIL1RN iMacs. **(I)** Heatmap showing the expression levels (by RNA-seq) of Interferon-related genes identified as differentially expressed at 3 hours of IL-1β treatment between CTRL and sgIL1RN iMacs. **(J)** Uniform Manifold Approximation and Projection (UMAP) plot depicting the cellular identities in the MoMac-Verse scRNA-seq dataset (*40*). **(K)** UMAP and balloon plot showing *IL1RN* expression across the MoMac-Verse. The cellular identities showing the highest *IL1RN* levels are highlighted in bold in the ballon plot and depicted in the UMAP. **(L)** Balloon plot showing scaled expression across the MoMac-Verse scRNA-seq dataset for the 0h-up and 0h-down gene signatures identified in (C). **(M)** Lollipop plot depicting the TF activity predicted from mRNA expression of target genes with DoRothEA v2.0 (*39*) in 16h ILβ-treated sgIL1RN and CTRL iMacs. Lollipop size indicates the total number of genes regulated by each transcriptional regulon.

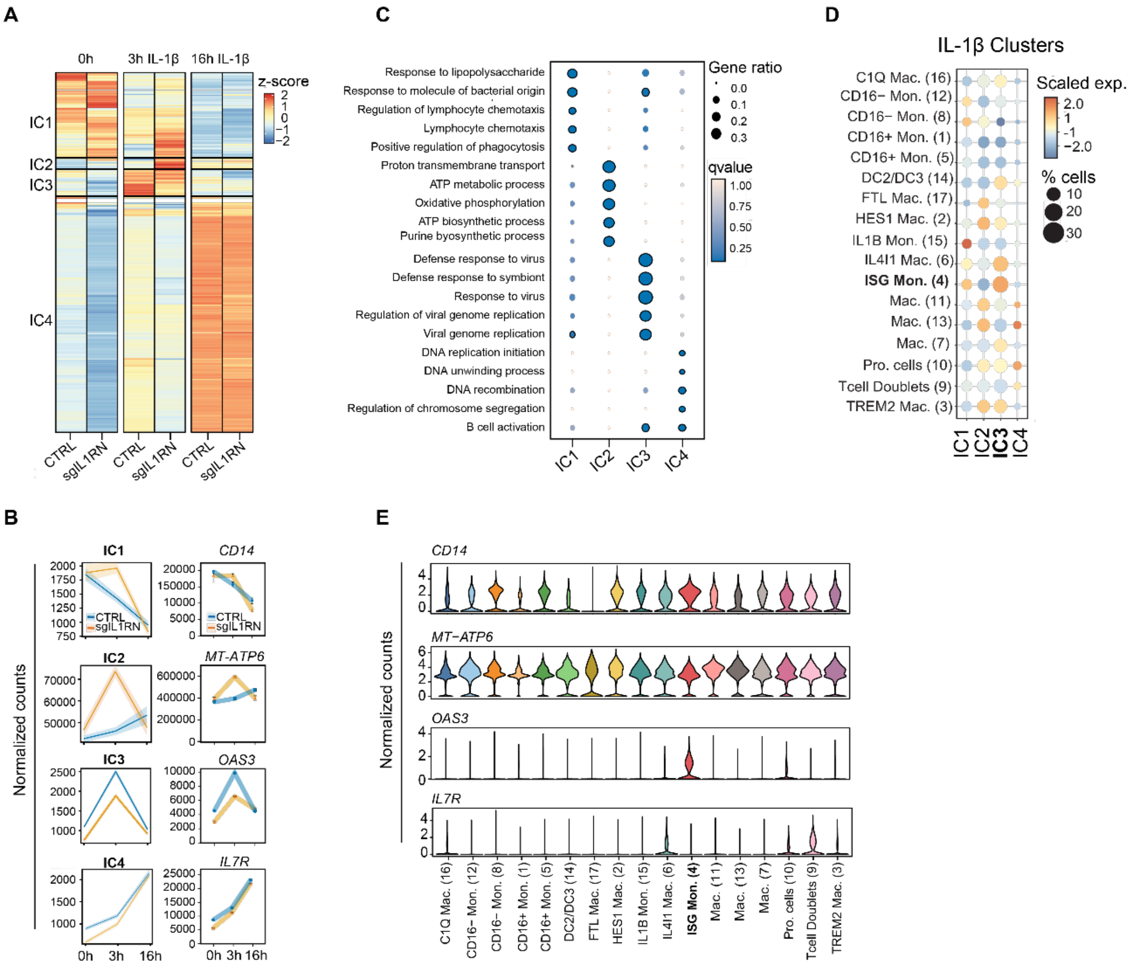

Fig. S7. Related to Fig. 5.
(A) Clustering of the transcriptional dynamics (by RNA-seq) observed during the IL-1β-treatment in sgIL1RN and CTRL iMacs. Average scaled normalized counts of differentially expressed genes (FDR<0.05) are represented at each timepoint (n=2 biologically independent replicates). Black lines show splitting by k-means clustering. IL-1β-treatment Clusters (IC1-4). **(B)** Left: quantification of RNA signal at the IL-1β-treatment clusters (IC1-4) in (A). Right: expression dynamics in sgIL1RN and CTRL iMacs of representative genes from the clusters. **(C)** Balloon plot depicting the Gene Ontology (GO) enrichment analysis for the genes associated with clusters in (A-B). The top 5 most significantly over-represented biological processes (BP) terms for each cluster are plotted. Ratio of genes of interest over all unique genes (GeneRatio) and qvalue are shown. Significant terms (qvalue<0.05) are highlighted with a black stroke. **(D)** Balloon plot showing scaled expression for the genes in the clusters from (A-B) across the MoMac-Verse scRNA-seq dataset. **(E)** Violin plots displaying the normalized expression of representative genes from the clusters in (A-B) across the MoMac-Verse scRNA-seq dataset (*40*).

Valcárcel_Supp. Fig 8

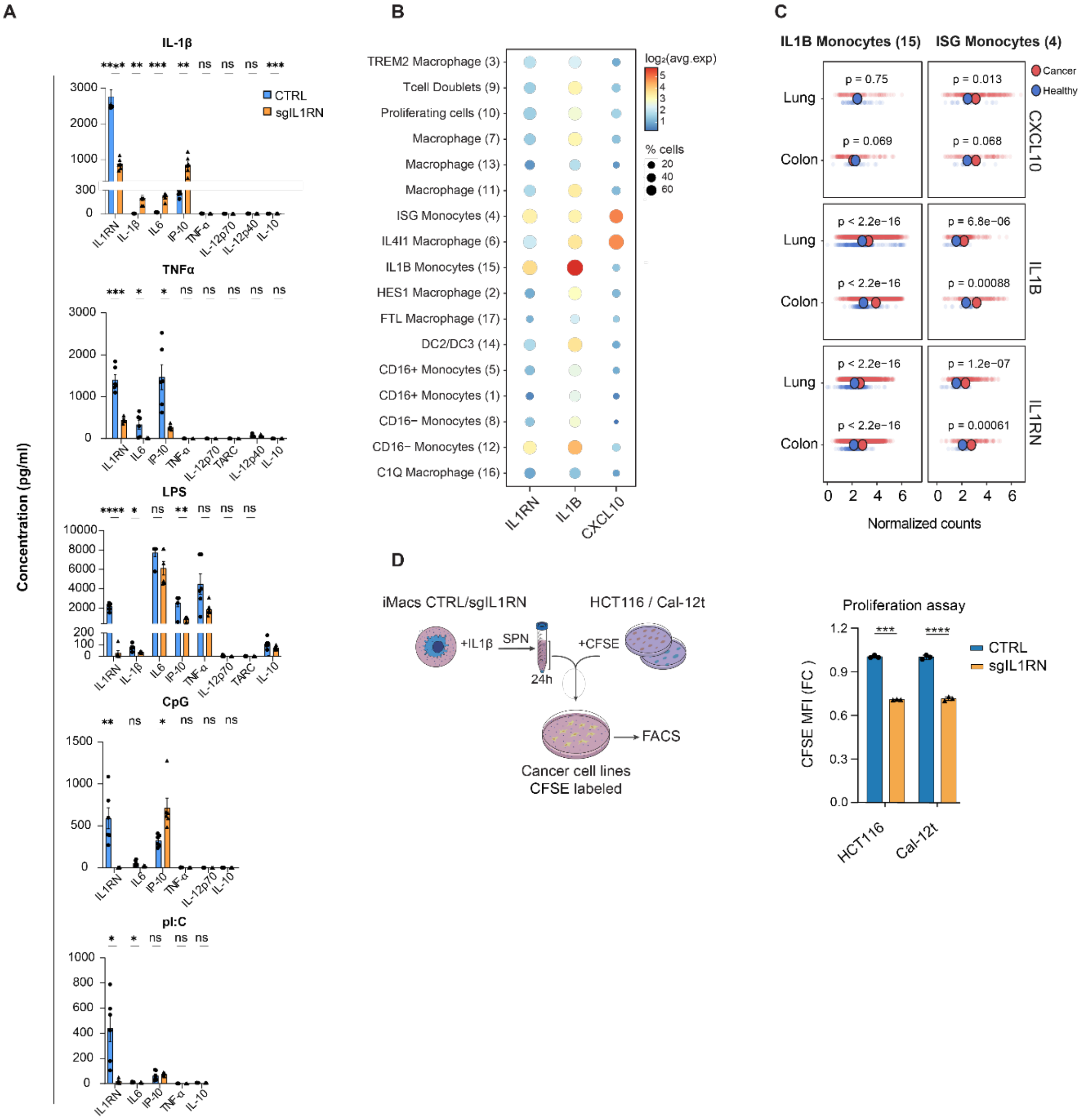

Fig. S8. Related to Fig. 5.

(A) Quantification of the concentration of the different cytokines measured by the LEGENDplex™ Human M1/M2 Macrophage Panel (10-plex) in the supernatant of sgIL1RN and CTRL iMacs in response to various inflammatory and pathogenic stimuli (IL-1β, TNFα, LPS, CpG, and pI:C). Unpaired two-tailed Student's t-test with Benjamini-Hochberg, n = 6 biologically independent samples per group, mean ± s.e.m., (*p<0.05; **p<0.01; ***p<0.001; ****p<0.0001). **(B)** Balloon plots showing *IL1B, IL1RN and CXCL10 (IP-10)* normalized expression levels across the MoMac-Verse scRNA-seq dataset **(C)** Balloon plots showing *CXCL10 (IP-10), IL1B and IL1RN* normalized expression levels in healthy and cancer IL1B Mon (15) and ISG-Mon (4) from lung and colon tissues from the MoMac-Verse scRNA-seq dataset. Average expression values for each group are shown as circles with a black stroke. P-values were calculated using a two-sided Wilcoxon rank-sum test. **(D)** Proliferation assay (by CFSE retention) assessing the impact of the differential cytokines produced by the sgIL1RN iMacs in (A) on the proliferation of HCT116 and Cal12-t cancer cell lines. Left: Schematic of the experimental setup. Right: Plot showing the normalized Mean Fluorescence Intensity (MFI) for the CFSE signal (4 days after staining) in cancer cells exposed to the supernatant of

sgIL1RN or the supernatant of CTRL iMacs. One-way Anova n=3, mean s.e.m., (***p< 0.001; ****p<0.0001).

SUPPLEMENTAL TABLES

Supplemental Table 1: MethylationEPIC array probes used in **Fig. S3G**.

Gene name	EPIC array probes
AIFL	cg1431702, cg14351708, cg26572165
CD244	cg17283691, cg13143320
CREB5	cg07660664
SCNN1A	cg17727262
TMBIM6	cg20081348
VWA8	cg07584516
TNFRSF1A	cg00574580, cg23039316
ARRB1	cg20581146
FA2H	cg11572506
LINC01851	cg01136754
LINC02292	cg2077388
RPH3AL	cg08770870, cg10440639,cg11940040,cg23246911
UICLM	cg101434416, cg12525096
VAC14	cg02529145, cg02643387, cg14813551
LINC02981	cg23096130

Supplemental Table 2: Oligonucleotides used to construct sgRNAs targeting dCas9 for methylation editing**.**

	Forward (5' to 3')	Reverse (5' to 3')
ILsgRNA_H1	TCCCACTCCATTGCGACACTTAGTG	CACTAAGTGTCGCAATGGAG
ILsgRNA_mU6	TTGTTTGCTTCTCGCAGTGGGGCAGGG	CCCTGCCCCACTGCGAGAAG
ILsgRNA_ hU6	CACCGAGCTTGGGTGAGTGACTATT	AATAGTCACTCACCCAAGCT
ILsgRNA_7SK	CCTCGCCACAACTCTGGGCCCGCAA	TTGCGGGCCCAGAGTTGTGG
CTRLsgRNA_H1	TCCCACCTAAGGTTAAGTCGCCCTC	AAACGATTCCAATTCAGCGGGAGT
CTRLsgRNA_mU6	TTGTTTGGCCCCCGGGGGAAAAATTT	AAACCCGGGGGCCCCCTTTTTAAACAA
CTRLsgRNA_ hU6	CACCGTAGTACTTTCAAGAGTCCA	AAACTATCATGAAAGTTCTCAGGTC
CTRLsgRNA_7SK	CCTCGCACTACCAGAGCTAACTCA	AAACCGTGATGGTCTCGATTGAGT

Supplemental Table 3: Myeloid surface marker panel for spectral cytometry analyses.

Master mix	Antibody	Fluorophore	Brand	Catalog #	Clone	uL/test
Viability	CD16 (Fc gamma RIII)	BUV496	BD Biosciences (BD OptiBuild)	612944	3G8	0.6
Post-Viability	CD185 (CXCR5)	BV750	BD Biosciences (BD OptiBuild)	569501	RF8B2	5
Chemokine	CD183 (CXCR3)	PECy7	BioLegend	353719	G025H7	5
	CD194 (CCR4, CCR-4)	BB700	BD Biosciences (BD OptiBuild)	566475	1G1	2.5

General	CD11b	APC	BD Biosciences (BD OptiBuild)	550019	ICRF44	2.5
	CD11c	BUV661	BD Biosciences (BD OptiBuild)	612968	B-ly6	2.5
	CD226 (DNAM-1)	BUV737	BD Biosciences (BD OptiBuild)	748428	DX11	1.25
	CD33 (Siglec-3)	BUV805	BD Biosciences (BD OptiBuild)	749018	P67.6	1.25
	CD40 (TNFRSF5)	BV650	Biolegend	334337	5C3	2.5
	CD62L (L-selectin)	SBB615	Bio-Rad	MCA1076GA	FMC46	1.25
	CD80 (B7-1)	RB780	BD Biosciences (BD OptiBuild)	755432	2D10.4	1.25
	CD81 (TAPA-1)	RY586	BD Biosciences (BD OptiBuild)	753220	JS-81	0.15
	CD86 (B7-2)	BV510	BD Biosciences (BD horizon)	563461	2331	2.5
	HLA-DR (HLADR)	APC-Fire 750	BioLegend	307657	L243	0.3
	CD25	PE-Fire700	Biolegend	356145	M-A251	2.5
	CD163	BB786	BD Biosciences (BD OptiBuild)	568221	Clone GHI/61	0.6
	CD206	Alexa Fluor 647	Biolegend	321116	Clone 1502	0.0375
	CD14	Spark Blue™ 550	Biolegend	367147	63D3	0.3

Supplemental Table 4: RT-qPCR primer sequences.

	**Forward (5' to 3')**	**Reverse (5' to 3')**
IL1RN	IDT Cat #228354241	IDT Cat #228354241
HPRT	GACCAGTCAACAGGGGACAT	CTGCATTGTTTTGCCAGTGT
B2M	AGGCTATCCAGCGTACTCCA	TCAATGTCGGATGGATGAAA
BRCA1	CTGCTCTGGGTAAAGTTCATTGG	TAAAGGACACTGTGAAGGCCC
CDK1	CACTTGGCTTCAAAGCTGGC	TGGGTATGGTAGATCCCGGC
LEF1	ATTCTTGGCAGAAGGTGGCA	GCAGCTGTCATTCTTGGACC
CD79A	CCTTAGTCATATTCCCCCAG	TTTAGAGGGAAGAAGAGTGG
HK2	GCTCAACCATGACCAAGTGC	AACTCTCCGTGTTCTGTCCC
PGK1	CTGGGCAAGGATGTTCTGTT	CACATGAAAGCGGAGGTTCT
ITGAM	GGGGTCTCCACTAAATATCTC	CTGACCTGATATTGATGCTG
CD14	GATTACATAAACTGTCAGAGGC	TCCATGGTCGATAAGTCTTC

Supplemental Table 5: Primers used to determine dCas9-TET1 enrichment by ChIP-qPCR in **Fig. 3B**.

	**Forward (5' to 3')**	**Reverse (5' to 3')**
IL1RN –5kb TSS	GCAGTCGGGGTTGGGGTAA	ACTCAGGCTAGCAGAAACCAA

IL1RN promoter	GGAGGGTATTTCCGCTTCTC	GCCTCTGCAGATTTCCATTC

Supplemental Table 6: Pyrosequencing primer sequences and genomic positions.

List of the genomic positions for the CpGs analyzed by pyrosequencing in **Fig.3C**.

CpG code	Genomic position hg38
#1	chr2:113,127,510-113,127,511
#2	chr2:113,127,517-113,127,518
#3	chr2:113,127,539-113,127,540
#4	chr2:113,127,589-113,127,590

Primers used to amplify the bisulfite-converted *IL1RN* promoter before conducting pyrosequencing analysis.

	Forward (5' to 3')	Reverse-bio (5' to 3')	Region (5' to 3') hg38
IL1RN promoter	AGTGGGGTTGAAAGTGACAAC	CAGAATGGAAATCTGCAGAGGCCTC	chr2:113,127,448-113,127,645

Primers used for the pyrosequencing analysis.

	Forward (5' to 3')
S1 IL1RN	GAAATGCGAGGAGGGTATTTCCGCTTCTCG
S2 IL1RN	CGCTTCTCGCAGTGGGGCAGGGTGGCAGACGC

REFERENCES AND NOTES

1. A. Bird, DNA methylation patterns and epigenetic memory. *Genes Dev.* **16**, 6–21 (2002).

2. M. M. Dawlaty, A. Breiling, T. Le, M. I. Barrasa, G. Raddatz, Q. Gao, B. E. Powell, A. W. Cheng, K. F. Faull, F. Lyko, R. Jaenisch, Loss of Tet enzymes compromises proper differentiation of embryonic stem cells. *Dev. Cell* **29**, 102–111 (2014).

3. J. Charlton, E. J. Jung, A. L. Mattei, N. Bailly, J. Liao, E. J. Martin, P. Giesselmann, B. Brändl, E. K. Stamenova, F.-J. Müller, E. Kiskinis, A. Gnirke, Z. D. Smith, A. Meissner, TETs compete with DNMT3 activity in pluripotent cells at thousands of methylated somatic enhancers. *Nat. Genet.* **52**, 819–827 (2020).

4. H.-Q. Dai, B.-A. Wang, L. Yang, J.-J. Chen, G.-C. Zhu, M.-L. Sun, H. Ge, R. Wang, D. L. Chapman, F. Tang, X. Sun, G.-L. Xu, TET-mediated DNA demethylation controls gastrulation by regulating Lefty–Nodal signalling. *Nature* **538**, 528–532 (2016).

5. G. Greve, G. Andrieux, P. Schlosser, N. Blagitko-Dorfs, U.-U. Rehman, T. Ma, D. Pfeifer, G. Heil, A. Neubauer, J. Krauter, M. Heuser, H. R. Salih, K. Döhner, H. Döhner, B. Hackanson, M. Boerries, M. Lübbert, In vivo kinetics of early, non-random methylome and transcriptome changes induced by DNA-hypomethylating treatment in primary AML blasts. *Leukemia* **37**, 1018–1027 (2023).

6. L. Holtzman, C. A. Gersbach, Editing the epigenome: Reshaping the genomic landscape. *Annu. Rev. Genomics Hum. Genet.* **19**, 43–71 (2018).

7. F. Fuks, P. J. Hurd, R. Deplus, T. Kouzarides, The DNA methyltransferases associate with HP1 and the SUV39H1 histone methyltransferase. *Nucleic Acids Res.* **31**, 2305–2312 (2003).

8. H. Li, T. Rauch, Z.-X. Chen, P. E. Szabó, A. D. Riggs, G. P. Pfeifer, The histone methyltransferase SETDB1 and the DNA methyltransferase DNMT3A interact directly and localize to promoters silenced in cancer cells. *J. Biol. Chem.* **281**, 19489–19500 (2006).

9. E. Viré, C. Brenner, R. Deplus, L. Blanchon, M. Fraga, C. Didelot, L. Morey, A. Van Eynde, D. Bernard, J.-M. Vanderwinden, M. Bollen, M. Esteller, L. Di Croce, Y. De Launoit, F. Fuks,

The Polycomb group protein EZH2 directly controls DNA methylation. *Nature* **439**, 871–874 (2006).

10. L. De La Rica, J. Rodríguez-Ubreva, M. García, A. B. Islam, J. M. Urquiza, H. Hernando, J. Christensen, K. Helin, C. Gómez-Vaquero, E. Ballestar, PU.1 target genes undergo Tet2-coupled demethylation and DNMT3b-mediated methylation in monocyte-to-osteoclast differentiation. *Genome Biol.* **14**, R99 (2013).

11. Y. Costa, J. Ding, T. W. Theunissen, F. Faiola, T. A. Hore, P. V. Shliaha, M. Fidalgo, A. Saunders, M. Lawrence, S. Dietmann, S. Das, D. N. Levasseur, Z. Li, M. Xu, W. Reik, J. C. R. Silva, J. Wang, NANOG-dependent function of TET1 and TET2 in establishment of pluripotency. *Nature* **495**, 370–374 (2013).

12. J. L. Sardina, S. Collombet, T. V. Tian, A. Gómez, B. Di Stefano, C. Berenguer, J. Brumbaugh, R. Stadhouders, C. Segura-Morales, M. Gut, I. G. Gut, S. Heath, S. Aranda, L. Di Croce, K. Hochedlinger, D. Thieffry, T. Graf, Transcription factors drive Tet2-mediated enhancer demethylation to reprogram cell fate. *Cell Stem Cell* **23**, 727–741.e9 (2018).

13. X. Zhang, J. Su, M. Jeong, M. Ko, Y. Huang, H. J. Park, A. Guzman, Y. Lei, Y.-H. Huang, A. Rao, W. Li, M. A. Goodell, DNMT3A and TET2 compete and cooperate to repress lineage-specific transcription factors in hematopoietic stem cells. *Nat. Genet.* **48**, 1014–1023 (2016).

14. I. F. López-Moyado, A. Tsagaratou, H. Yuita, H. Seo, B. Delatte, S. Heinz, C. Benner, A. Rao, Paradoxical association of TET loss of function with genome-wide DNA hypomethylation. *Proc. Natl. Acad. Sci. U.S.A.* **116**, 16933–16942 (2019).

15. J. Stomper, J. C. Rotondo, G. Greve, M. Lübbert, Hypomethylating agents (HMA) for the treatment of acute myeloid leukemia and myelodysplastic syndromes: Mechanisms of resistance and novel HMA-based therapies. *Leukemia* **35**, 1873–1889 (2021).

16. A. De Mendoza, T. V. Nguyen, E. Ford, D. Poppe, S. Buckberry, J. Pflueger, M. R. Grimmer, S. Stolzenburg, O. Bogdanovic, A. Oshlack, P. J. Farnham, P. Blancafort, R. Lister, Large-scale manipulation of promoter DNA methylation reveals context-specific transcriptional responses and stability. *Genome Biol.* **23**, 163 (2022).

17. C. Policarpi, J. Dabin, J. A. Hackett, Epigenetic editing: Dissecting chromatin function in context. *Bioessays* **43**, e2000316 (2021).

18. C. Policarpi, M. Munafò, S. Tsagkris, V. Carlini, J. A. Hackett, Systematic epigenome editing captures the context-dependent instructive function of chromatin modifications. *Nat. Genet.* **56**, 1168–1180 (2024).

19. Y. Yin, E. Morgunova, A. Jolma, E. Kaasinen, B. Sahu, S. Khund-Sayeed, P. K. Das, T. Kivioja, K. Dave, F. Zhong, K. R. Nitta, M. Taipale, A. Popov, P. A. Ginno, S. Domcke, J. Yan, D. Schübeler, C. Vinson, J. Taipale, Impact of cytosine methylation on DNA binding specificities of human transcription factors. *Science* **356**, eaaj2239 (2017).

20. A. Monteagudo-Sánchez, J. Richard Albert, M. Scarpa, D. Noordermeer, M. V. C. Greenberg, The impact of the embryonic DNA methylation program on CTCF-mediated genome regulation. *Nucleic Acids Res.* **52**, 10934–10950 (2024).

21. D. Álvarez-Errico, R. Vento-Tormo, M. Sieweke, E. Ballestar, Epigenetic control of myeloid cell differentiation, identity and function. *Nat. Rev. Immunol.* **15**, 7–17 (2015).

22. A. Amabile, A. Migliara, P. Capasso, M. Biffi, D. Cittaro, L. Naldini, A. Lombardo, Inheritable silencing of endogenous genes by hit-and-run targeted epigenetic editing. *Cell* **167**, 219–232.e14 (2016).

23. E. A. Saunderson, H. H. Encabo, J. Devis, K. Rouault-Pierre, M. Piganeau, C. G. Bell, J. G. Gribben, D. Bonnet, G. Ficz, CRISPR/dCas9 DNA methylation editing is heritable during human hematopoiesis and shapes immune progeny. *Proc. Natl. Acad. Sci. U.S.A.* **120**, e2300224120 (2023).

24. F. Rapino, E. F. Robles, J. A. Richter-Larrea, E. M. Kallin, J. A. Martinez-Climent, T. Graf, C/EBPα induces highly efficient macrophage transdifferentiation of B lymphoma and leukemia cell lines and impairs their tumorigenicity. *Cell Rep.* **3**, 1153–1163 (2013).

25. M. M. Gaidt, T. S. Ebert, D. Chauhan, T. Schmidt, J. L. Schmid-Burgk, F. Rapino, A. A. B. Robertson, M. A. Cooper, T. Graf, V. Hornung, Human monocytes engage an alternative inflammasome pathway. *Immunity* **44**, 833–846 (2016).

26. G. Stik, E. Vidal, M. Barrero, S. Cuartero, M. Vila-Casadesús, J. Mendieta-Esteban, T. V. Tian, J. Choi, C. Berenguer, A. Abad, B. Borsari, F. Le Dily, P. Cramer, M. A. Marti-Renom, R. Stadhouders, T. Graf, CTCF is dispensable for immune cell transdifferentiation but facilitates an acute inflammatory response. *Nat. Genet.* **52**, 655–661 (2020).

27. J. Choi, K. Lysakovskaia, G. Stik, C. Demel, J. Söding, T. V. Tian, T. Graf, P. Cramer, Evidence for additive and synergistic action of mammalian enhancers during cell fate determination. *eLife* **10**, e65381 (2021).

28. G. C. Hon, C.-X. Song, T. Du, F. Jin, S. Selvaraj, A. Y. Lee, C. Yen, Z. Ye, S.-Q. Mao, B.-A. Wang, S. Kuan, L. E. Edsall, B. S. Zhao, G.-L. Xu, C. He, B. Ren, 5mC oxidation by Tet2 modulates enhancer activity and timing of transcriptome reprogramming during differentiation. *Mol. Cell* **56**, 286–297 (2014).

29. C. Gabay, C. Lamacchia, G. Palmer, IL-1 pathways in inflammation and human diseases. *Nat. Rev. Rheumatol.* **6**, 232–241 (2010).

30. S. H. Stricker, A. Köferle, S. Beck, From profiles to function in epigenomics. *Nat. Rev. Genet.* **18**, 51–66 (2017).

31. X. S. Liu, H. Wu, X. Ji, Y. Stelzer, X. Wu, S. Czauderna, J. Shu, D. Dadon, R. A. Young, R. Jaenisch, Editing DNA methylation in the mammalian genome. *Cell* **167**, 233–247.e17 (2016).

32. X. S. Liu, H. Wu, M. Krzisch, X. Wu, J. Graef, J. Muffat, D. Hnisz, C. H. Li, B. Yuan, C. Xu, Y. Li, D. Vershkov, A. Cacace, R. A. Young, R. Jaenisch, Rescue of fragile X syndrome neurons by DNA methylation editing of the FMR1 gene. *Cell* **172**, 979–992.e6 (2018).

33. C. Pflueger, D. Tan, T. Swain, T. Nguyen, J. Pflueger, C. Nefzger, J. M. Polo, E. Ford, R. Lister, A modular dCas9-SunTag DNMT3A epigenome editing system overcomes pervasive

off-target activity of direct fusion dCas9-DNMT3A constructs. *Genome Res.* **28**, 1193–1206 (2018).

34. C. Galonska, J. Charlton, A. L. Mattei, J. Donaghey, K. Clement, H. Gu, A. W. Mohammad, E. K. Stamenova, D. Cacchiarelli, S. Klages, B. Timmermann, T. Cantz, H. R. Schöler, A. Gnirke, M. J. Ziller, A. Meissner, Genome-wide tracking of dCas9-methyltransferase footprints. *Nat. Commun.* **9**, 597 (2018).

35. K. C. Higa, A. Goodspeed, J. S. Chavez, M. De Dominici, E. Danis, V. Zaberezhnyy, J. L. Rabe, D. G. Tenen, E. M. Pietras, J. DeGregori, Chronic interleukin-1 exposure triggers selection for *Cebpa*-knockout multipotent hematopoietic progenitors. *J. Exp. Med.* **218**, e20200560 (2021).

36. A. Villatoro, V. Cuminetti, A. Bernal, C. Torroja, I. Cossío, A. Benguría, M. Ferré, J. Konieczny, E. Vázquez, A. Rubio, P. Utnes, A. Tello, X. You, C. G. Fenton, R. H. Paulssen, J. Zhang, F. Sánchez-Cabo, A. Dopazo, A. Vik, E. Anderssen, A. Hidalgo, L. Arranz, Endogenous IL-1 receptor antagonist restricts healthy and malignant myeloproliferation. *Nat. Commun.* **14**, 12 (2023).

37. D.-E. Zhang, P. Zhang, N.-d. Wang, C. J. Hetherington, G. J. Darlington, D. G. Tenen, Absence of granulocyte colony-stimulating factor signaling and neutrophil development in CCAAT enhancer binding protein α-deficient mice. *Proc. Natl. Acad. Sci. U.S.A.* **94**, 569–574 (1997).

38. W. P. Arend, H. G. Welgus, R. C. Thompson, S. P. Eisenberg, Biological properties of recombinant human monocyte-derived interleukin 1 receptor antagonist. *J. Clin. Invest.* **85**, 1694–1697 (1990).

39. L. Garcia-Alonso, C. H. Holland, M. M. Ibrahim, D. Turei, J. Saez-Rodriguez, Benchmark and integration of resources for the estimation of human transcription factor activities. *Genome Res.* **29**, 1363–1375 (2019).

40. K. Mulder, A. A. Patel, W. T. Kong, C. Piot, E. Halitzki, G. Dunsmore, S. Khalilnezhad, S. E. Irac, A. Dubuisson, M. Chevrier, X. M. Zhang, J. K. C. Tam, T. K. H. Lim, R. M. M. Wong,

R. Pai, A. I. S. Khalil, P. K. H. Chow, S. Z. Wu, G. Al-Eryani, D. Roden, A. Swarbrick, J. K. Y. Chan, S. Albani, L. Derosa, L. Zitvogel, A. Sharma, J. Chen, A. Silvin, A. Bertoletti, C. Blériot, C.-A. Dutertre, F. Ginhoux, Cross-tissue single-cell landscape of human monocytes and macrophages in health and disease. *Immunity* **54**, 1883–1900.e5 (2021).

41. M. Gaestel, A. Kotlyarov, M. Kracht, Targeting innate immunity protein kinase signalling in inflammation. *Nat. Rev. Drug Discov.* **8**, 480–499 (2009).

42. B. Di Stefano, S. Collombet, J. S. Jakobsen, M. Wierer, J. L. Sardina, A. Lackner, R. Stadhouders, C. Segura-Morales, M. Francesconi, F. Limone, M. Mann, B. Porse, D. Thieffry, T. Graf, C/EBPα creates elite cells for iPSC reprogramming by upregulating Klf4 and increasing the levels of Lsd1 and Brd4. *Nat. Cell Biol.* **18**, 371–381 (2016).

43. O. Morante-Palacios, L. Ciudad, R. Micheroli, C. de la Calle-Fabregat, T. Li, G. Barbisan, M. Houtman, S. G. Edalat, M. Frank-Bertoncelj, C. Ospelt, E. Ballestar, Coordinated glucocorticoid receptor and MAFB action induces tolerogenesis and epigenome remodeling in dendritic cells. *Nucleic Acids Res.* **50**, 108–126 (2022).

44. A. Lazarenkov, J. L. Sardina, Dissecting *TET2* regulatory networks in blood differentiation and cancer. *Cancers* **14**, 830 (2022).

45. E. M. Kallin, J. Rodríguez-Ubreva, J. Christensen, L. Cimmino, I. Aifantis, K. Helin, E. Ballestar, T. Graf, Tet2 facilitates the derepression of myeloid target genes during CEBPα-induced transdifferentiation of pre-B cells. *Mol. Cell* **48**, 266–276 (2012).

46. K. Takahashi, S. Yamanaka, Induction of pluripotent stem cells from mouse embryonic and adult fibroblast cultures by defined factors. *Cell* **126**, 663–676 (2006).

47. L. Mosteiro, C. Pantoja, N. Alcazar, R. M. Marión, D. Chondronasiou, M. Rovira, P. J. Fernandez-Marcos, M. Muñoz-Martin, C. Blanco-Aparicio, J. Pastor, G. Gómez-López, A. De Martino, M. A. Blasco, M. Abad, M. Serrano, Tissue damage and senescence provide critical signals for cellular reprogramming in vivo. *Science* **354**, aaf4445 (2016).

48. B. Di Stefano, J. L. Sardina, C. Van Oevelen, S. Collombet, E. M. Kallin, G. P. Vicent, J. Lu, D. Thieffry, M. Beato, T. Graf, C/EBPα poises B cells for rapid reprogramming into induced pluripotent stem cells. *Nature* **506**, 235–239 (2014).

49. M. Plana-Carmona, G. Stik, R. Bulteau, C. Segura-Morales, N. Alcázar, C. D. R. Wyatt, A. Klonizakis, L. De Andrés-Aguayo, M. Gasnier, T. V. Tian, G. Torcal Garcia, M. Vila-Casadesús, N. Plachta, M. Serrano, M. Francesconi, T. Graf, The trophectoderm acts as a niche for the inner cell mass through C/EBPα-regulated IL-6 signaling. *Stem Cell Reports* **17**, 1991–2004 (2022).

50. H. Xie, M. Ye, R. Feng, T. Graf, Stepwise reprogramming of B cells into macrophages. *Cell* **117**, 663–676 (2004).

51. C. A. Dinarello, The IL-1 family of cytokines and receptors in rheumatic diseases. *Nat. Rev. Rheumatol.* **15**, 612–632 (2019).

52. F. Grebien, M. Vedadi, M. Getlik, R. Giambruno, A. Grover, R. Avellino, A. Skucha, S. Vittori, E. Kuznetsova, D. Smil, D. Barsyte-Lovejoy, F. Li, G. Poda, M. Schapira, H. Wu, A. Dong, G. Senisterra, A. Stukalov, K. V. M. Huber, A. Schönegger, R. Marcellus, M. Bilban, C. Bock, P. J. Brown, J. Zuber, K. L. Bennett, R. Al-awar, R. Delwel, C. Nerlov, C. H. Arrowsmith, G. Superti-Furga, Pharmacological targeting of the Wdr5-MLL interaction in C/EBPα N-terminal leukemia. *Nat. Chem. Biol.* **11**, 571–578 (2015).

53. G. Cavalli, C. A. Dinarello, Anakinra therapy for non-cancer inflammatory diseases. *Front. Pharmacol.* **9**, 1157 (2018).

54. T. Huet, H. Beaussier, O. Voisin, S. Jouveshomme, G. Dauriat, I. Lazareth, E. Sacco, J.-M. Naccache, Y. Bézie, S. Laplanche, A. Le Berre, J. Le Pavec, S. Salmeron, J. Emmerich, J.-J. Mourad, G. Chatellier, G. Hayem, Anakinra for severe forms of COVID-19: A cohort study. *Lancet Rheumatol.* **2**, e393–e400 (2020).

55. I. Aksentijevich, S. L. Masters, P. J. Ferguson, P. Dancey, J. Frenkel, A. van Royen-Kerkhoff, R. Laxer, U. Tedgård, E. W. Cowen, T.-H. Pham, M. Booty, J. D. Estes, N. G. Sandler, N. Plass, D. L. Stone, M. L. Turner, S. Hill, J. A. Butman, R. Schneider, P. Babyn, H. I. El-

Shanti, E. Pope, K. Barron, X. Bing, A. Laurence, C.-C. R. Lee, D. Chapelle, G. I. Clarke, K. Ohson, M. Nicholson, M. Gadina, B. Yang, B. D. Korman, P. K. Gregersen, P. M. van Hagen, A. E. Hak, M. Huizing, P. Rahman, D. C. Douek, E. F. Remmers, D. L. Kastner, R. Goldbach-Mansky, An autoinflammatory disease with deficiency of the interleukin-1-receptor antagonist. *N. Engl. J. Med.* **360**, 2426–2437 (2009).

56. R. Vento-Tormo, D. Álvarez-Errico, A. Garcia-Gomez, J. Hernández-Rodríguez, S. Buján, M. Basagaña, M. Méndez, J. Yagüe, M. Juan, J. I. Aróstegui, E. Ballestar, DNA demethylation of inflammasome-associated genes is enhanced in patients with cryopyrin-associated periodic syndromes. *J. Allergy Clin. Immunol.* **139**, 202–211.e6 (2017).

57. U.S. Food and Drug Administration. "FDA Approves First Gene Therapies to Treat Patients with Sickle Cell Disease," FDA, 8 December 2023. https://www.fda.gov/news-events/press-announcements/fda-approves-first-gene-therapies-treat-patients-sickle-cell-disease.

58. L. Villiger, J. Joung, L. Koblan, J. Weissman, O. O. Abudayyeh, J. S. Gootenberg, CRISPR technologies for genome, epigenome and transcriptome editing. *Nat. Rev. Mol. Cell Biol.* **25**, 464–487 (2024).

59. K. D. Mayer-Barber, B. Yan, Clash of the cytokine titans: Counter-regulation of interleukin-1 and type I interferon-mediated inflammatory responses. *Cell. Mol. Immunol.* **14**, 22–35 (2017).

60. K. D. Mayer-Barber, B. B. Andrade, S. D. Oland, E. P. Amaral, D. L. Barber, J. Gonzales, S. C. Derrick, R. Shi, N. P. Kumar, W. Wei, X. Yuan, G. Zhang, Y. Cai, S. Babu, M. Catalfamo, A. M. Salazar, L. E. Via, C. E. Barry Iii, A. Sher, Host-directed therapy of tuberculosis based on interleukin-1 and type I interferon crosstalk. *Nature* **511**, 99–103 (2014).

61. S. I. Grivennikov, F. R. Greten, M. Karin, Immunity, inflammation, and cancer. *Cell* **140**, 883–899 (2010).

62. J. Qian, X. Guan, B. Xie, C. Xu, J. Niu, X. Tang, C. H. Li, H. M. Colecraft, R. Jaenisch, X. S. Liu, Multiplex epigenome editing of *MECP2* to rescue Rett syndrome neurons. *Sci. Transl. Med.* **15**, eadd4666 (2023).

63. M. A. Cappelluti, V. Mollica Poeta, S. Valsoni, P. Quarato, S. Merlin, I. Merelli, A. Lombardo, Durable and efficient gene silencing in vivo by hit-and-run epigenome editing. *Nature* **627**, 416–423 (2024).

64. C. Engler, R. Kandzia, S. Marillonnet, A one pot, one step, precision cloning method with high throughput capability. *PLOS ONE* **3**, e3647 (2008).

65. A. M. Kabadi, D. G. Ousterout, I. B. Hilton, C. A. Gersbach, Multiplex CRISPR/Cas9-based genome engineering from a single lentiviral vector. *Nucleic Acids Res.* **42**, e147 (2014).

66. C. Engler, S. Marillonnet, "Golden gate cloning" in *DNA Cloning and Assembly Methods*, S. Valla, R. Lale, Eds. (Humana Press, 2014; https://link.springer.com/10.1007/978-1-62703-764-8_9) vol. 1116 of *Methods in Molecular Biology*, pp. 119–131.

67. W. Huber, V. J. Carey, R. Gentleman, S. Anders, M. Carlson, B. S. Carvalho, H. C. Bravo, S. Davis, L. Gatto, T. Girke, R. Gottardo, F. Hahne, K. D. Hansen, R. A. Irizarry, M. Lawrence, M. I. Love, J. MacDonald, V. Obenchain, A. K. Oleś, H. Pagès, A. Reyes, P. Shannon, G. K. Smyth, D. Tenenbaum, L. Waldron, M. Morgan, Orchestrating high-throughput genomic analysis with Bioconductor. *Nat. Methods* **12**, 115–121 (2015).

68. The ENCODE Project Consortium, An integrated encyclopedia of DNA elements in the human genome. *Nature* **489**, 57–74 (2012).

69. G. Yu, L.-G. Wang, Q.-Y. He, ChIPseeker: An R/Bioconductor package for ChIP peak annotation, comparison and visualization. *Bioinformatics* **31**, 2382–2383 (2015).

70. G. Yu, L.-G. Wang, Y. Han, Q.-Y. He, clusterProfiler: An R package for comparing biological themes among gene clusters. *OMICS* **16**, 284–287 (2012).

71. M. Milacic, D. Beavers, P. Conley, C. Gong, M. Gillespie, J. Griss, R. Haw, B. Jassal, L. Matthews, B. May, R. Petryszak, E. Ragueneau, K. Rothfels, C. Sevilla, V. Shamovsky, R. Stephan, K. Tiwari, T. Varusai, J. Weiser, A. Wright, G. Wu, L. Stein, H. Hermjakob, P. D'Eustachio, The reactome pathway knowledgebase 2024. *Nucleic Acids Res.* **52**, D672–D678 (2024).

72. A. Subramanian, P. Tamayo, V. K. Mootha, S. Mukherjee, B. L. Ebert, M. A. Gillette, A. Paulovich, S. L. Pomeroy, T. R. Golub, E. S. Lander, J. P. Mesirov, Gene set enrichment analysis: A knowledge-based approach for interpreting genome-wide expression profiles. *Proc. Natl. Acad. Sci. U.S.A.* **102**, 15545–15550 (2005).

73. G. Korotkevich, V. Sukhov, N. Budin, B. Shpak, M. N. Artyomov, A. Sergushichev, Fast gene set enrichment analysis. bioRxiv 060012 [Preprint] (2016). https://doi.org/10.1101/060012.

74. F. Ramírez, F. Dündar, S. Diehl, B. A. Grüning, T. Manke, deepTools: A flexible platform for exploring deep-sequencing data. *Nucleic Acids Res.* **42**, W187–W191 (2014).

75. S. Heinz, C. Benner, N. Spann, E. Bertolino, Y. C. Lin, P. Laslo, J. X. Cheng, C. Murre, H. Singh, C. K. Glass, Simple combinations of lineage-determining transcription factors prime cis-regulatory elements required for macrophage and B cell identities. *Mol. Cell* **38**, 576–589 (2010).

76. A. R. Quinlan, I. M. Hall, BEDTools: A flexible suite of utilities for comparing genomic features. *Bioinformatics* **26**, 841–842 (2010).

77. S. Müller-Dott, E. Tsirvouli, M. Vazquez, R. O. Ramirez Flores, P. Badia-i-Mompel, R. Fallegger, D. Türei, A. Lægreid, J. Saez-Rodriguez, Expanding the coverage of regulons from high-confidence prior knowledge for accurate estimation of transcription factor activities. *Nucleic Acids Res.* **51**, 10934–10949 (2023).

78. P. Badia-i-Mompel, J. Vélez Santiago, J. Braunger, C. Geiss, D. Dimitrov, S. Müller-Dott, P. Taus, A. Dugourd, C. H. Holland, R. O. Ramirez Flores, J. Saez-Rodriguez, decoupleR: Ensemble of computational methods to infer biological activities from omics data. *Bioinformatics Advances* **2**, vbac016 (2022).

79. Complex heatmap visualization - Gu - 2022 - iMeta - Wiley Online Library. https://onlinelibrary.wiley.com/doi/full/10.1002/imt2.43, 1, e43.

80. M. E. Ritchie, B. Phipson, D. Wu, Y. Hu, C. W. Law, W. Shi, G. K. Smyth, *limma* powers differential expression analyses for RNA-sequencing and microarray studies. *Nucleic Acids Res.* **43**, e47 (2015).

81. J. R. Conway, A. Lex, N. Gehlenborg, UpSetR: An R package for the visualization of intersecting sets and their properties. *Bioinformatics* **33**, 2938–2940 (2017).

82. M. I. Love, W. Huber, S. Anders, Moderated estimation of fold change and dispersion for RNA-seq data with DESeq2. *Genome Biol.* **15**, 550 (2014).

83. A. M. Bolger, M. Lohse, B. Usadel, Trimmomatic: A flexible trimmer for Illumina sequence data. *Bioinformatics* **30**, 2114–2120 (2014).

84. F. Krueger, S. R. Andrews, Bismark: A flexible aligner and methylation caller for bisulfite-seq applications. *Bioinformatics* **27**, 1571–1572 (2011).

85. M. J. Aryee, A. E. Jaffe, H. Corrada-Bravo, C. Ladd-Acosta, A. P. Feinberg, K. D. Hansen, R. A. Irizarry, Minfi: A flexible and comprehensive Bioconductor package for the analysis of Infinium DNA methylation microarrays. *Bioinformatics* **30**, 1363–1369 (2014).

86. A. Dobin, C. A. Davis, F. Schlesinger, J. Drenkow, C. Zaleski, S. Jha, P. Batut, M. Chaisson, T. R. Gingeras, STAR: Ultrafast universal RNA-seq aligner. *Bioinformatics* **29**, 15–21 (2013).

87. Y. Liao, G. K. Smyth, W. Shi, featureCounts: An efficient general purpose program for assigning sequence reads to genomic features. *Bioinformatics* **30**, 923–930 (2014).

88. Y. Hao, S. Hao, E. Andersen-Nissen, W. M. Mauck III, S. Zheng, A. Butler, M. J. Lee, A. J. Wilk, C. Darby, M. Zager, P. Hoffman, M. Stoeckius, E. Papalexi, E. P. Mimitou, J. Jain, A. Srivastava, T. Stuart, L. M. Fleming, B. Yeung, A. J. Rogers, J. M. McElrath, C. A. Blish, R. Gottardo, P. Smibert, R. Satija, Integrated analysis of multimodal single-cell data. *Cell* **184**, 3573–3587.e29 (2021).

89. B. Langmead, S. L. Salzberg, Fast gapped-read alignment with Bowtie 2. *Nat. Methods* **9**, 357–359 (2012).

90. Y. Chen, L. Chen, A. T. L. Lun, P. L. Baldoni, G. K. Smyth, edgeR v4: Powerful differential analysis of sequencing data with expanded functionality and improved support for small counts and larger datasets. *Nucleic Acids Res.* **53**, gkaf018 (2025).

91. M. S. Hasemann, F. K. B. Lauridsen, J. Waage, J. S. Jakobsen, A.-K. Frank, M. B. Schuster, N. Rapin, F. O. Bagger, P. S. Hoppe, T. Schroeder, B. T. Porse, C/EBPα is required for long-term self-renewal and lineage priming of hematopoietic stem cells and for the maintenance of epigenetic configurations in multipotent progenitors. *PLOS Genet.* **10**, e1004079 (2014).

92. D. Lara-Astiaso, A. Weiner, E. Lorenzo-Vivas, I. Zaretsky, D. A. Jaitin, E. David, H. Keren-Shaul, A. Mildner, D. Winter, S. Jung, N. Friedman, I. Amit, Chromatin state dynamics during blood formation. *Science* **345**, 943–949 (2014).
